# Mechanochemical Oxidative Degradation of Thienopyridine Containing Drugs: Toward a Simple Tool for the Prediction of Drug Stability

**DOI:** 10.1021/acscentsci.3c00167

**Published:** 2023-05-16

**Authors:** Everaldo F. Krake, Laura Backer, Benjamin Andres, Wolfgang Baumann, Norbert Handler, Helmut Buschmann, Ulrike Holzgrabe, Carsten Bolm, Torsten Beweries

**Affiliations:** † Leibniz-Institut für Katalyse, e.V., Albert-Einstein-Strasse 29a, 18059 Rostock, Germany; ‡ Institut für Pharmazie und Lebensmittelchemie,Universität Würzburg, Am Hubland, 97074 Würzburg Germany; § RD&C Research, Development & Consulting GmbH, Neuwaldegger Strasse 35/2/3, 1170 Vienna, Austria; ∥ Institut für Organische Chemie, RWTH Aachen University, Landoltweg 1, 52074 Aachen, Germany

## Abstract

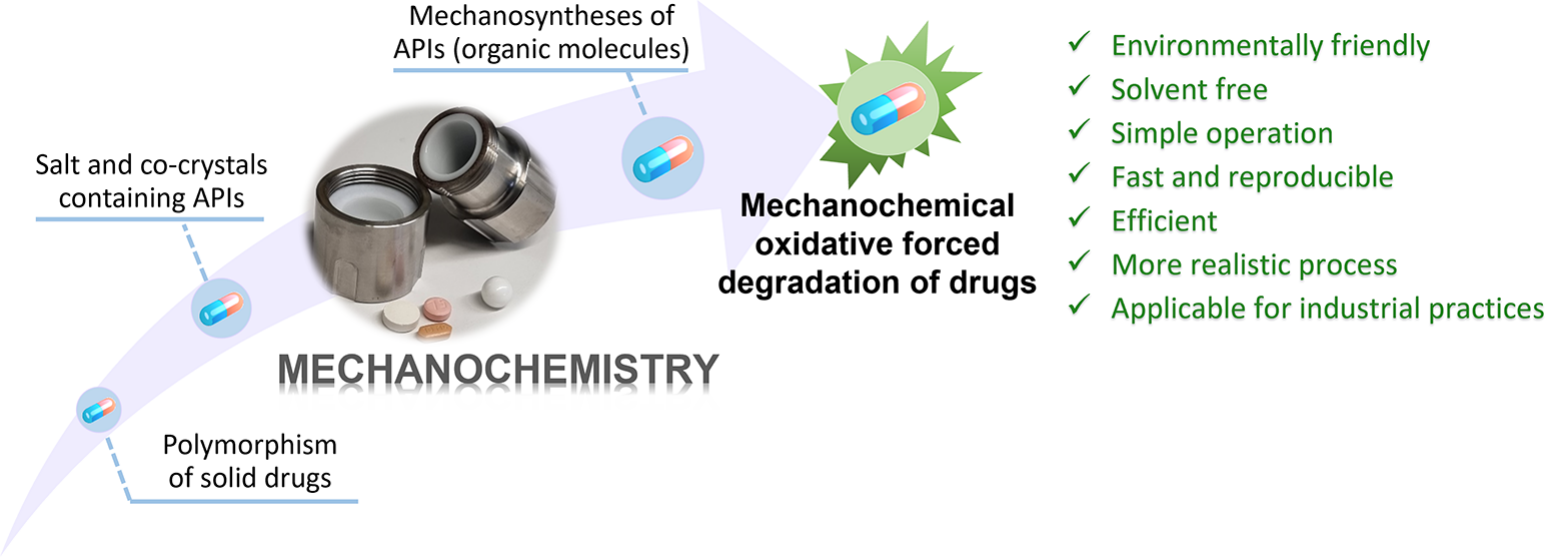

The long-term stability of an active-pharmaceutical ingredient and its drug products plays an important role in the licensing process of new pharmaceuticals and for the application of the drug at the patient. It is, however, difficult to predict degradation profiles at early stages of the development of new drugs, making the entire process very time-consuming and costly. Forced mechanochemical degradation under controlled conditions can be used to realistically model long-term degradation processes naturally occurring in drug products, avoiding the use of solvents, thus excluding irrelevant solution-based degradation pathways. We present the forced mechanochemical oxidative degradation of three platelet inhibitor drug products, where the drug products contain thienopyridine. Model studies using clopidogrel hydrogen sulfate (**CLP**) and its drug formulation Plavix show that the controlled addition of excipients does not affect the nature of the main degradants. Experiments using drug products Ticlopidin-neuraxpharm and Efient show that significant degradation occurs after short reaction times of only 15 min. These results highlight the potential of mechanochemistry for the study of degradation processes of small molecules relevant to the prediction of degradation profiles during the development of new drugs. Furthermore, these data provide exciting insights into the role of mechanochemistry for chemical synthesis in general.

## Abstract 2

Mechanochemical oxidative forced degradation is a realistic tool for predicting drug products stability, avoiding the use of solvents, and excluding irrelevant solution-based degradation pathways.

## Introduction

**Figure 1:**
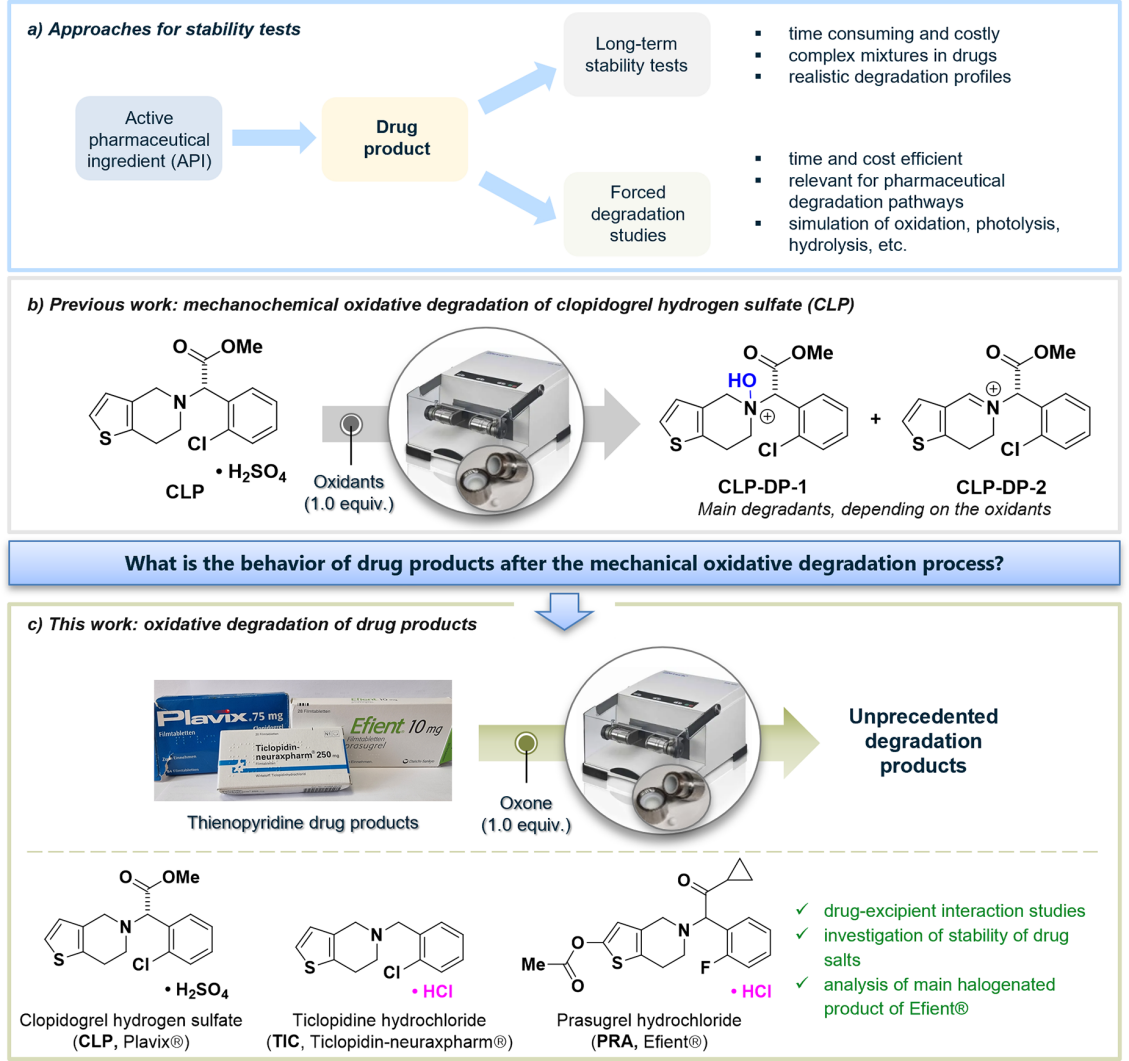
Contextualization of this study.

The approval of commercial pharmaceutical products requires the investigation and disclosure of stability data at regulatory authorities such as the European Medicines Agency (EMA) or the US Food and Drugs Administration (FDA).^1,2^ Such studies are, however, complicated by the fact that virtually all drugs are multicomponent mixtures, containing the active pharmaceutical ingredient (API) and various excipients, otherwise known as drug carriers, in various solid (powders, tablets, granules), liquid (solution, suspension, syrup), and semisolid forms (gels, creams).^3^ Due to the complexity of the systems, those studies are typically very time-consuming as degradation profiles and kinetics are unique for each pharmaceutical product, depending not only on the composition of the drug but also on the exact structures of the API (Figure 1a). For example, the presence of different counteranions or solvates can have a substantial effect on the kinetics and nature of thermal and photochemical degradation processes.^4,5^ The way in which stability studies are conducted, relevant thresholds for testing impurities, and a more flexible approach to pharmaceutical quality based on Good Manufacturing Practice (GMP) and risk management have been laid down by the International Council for Harmonisation of Technical Requirements for Pharmaceuticals for Human Use (ICH). Monitoring the long-term stability is possible by simply storing the substances or drug products under controlled conditions at different temperature and humidity for a long period of time. As an alternative, prediction of long-term stability and identification of degradation products was to date mostly done using stress tests in aqueous acidic, neutral, or alkaline solution under defined thermal, photochemical, or oxidative conditions.^6^ Naturally, studies under these conditions cannot reflect the reality of naturally occurring solid drug decomposition in all cases, and such prediction methods are known to result in high failure rates as nonrelevant degradants may be formed.^7^ Forced degradation testing is required by the ICH guidelines applying harsher conditions than that for accelerated stability studies (higher temperatures and humidity, various environments). Ideally, all possible degradation products should be produced, even though they might not be present on the long run at storage conditions. Due to the use of specific forced degradation conditions (high temperature and humidity, solvents, pH, etc.) not all degradation products might eventually be relevant, especially the use of solvents and aqueous solutions (e.g., 3% H_2_O_2_ or similar) might lead to degradation pathways and products which are not representative for a solid drug formulation. However, forced degradation studies are an essential part of the drug development process, and in addition, they deliver very helpful and versatile data for the development and justification of a stability indicating analytical method. Hence, further improvement of the applicability and predictability especially for solid drug systems is highly desired.

Embracing the enormous potential of mechanochemistry for various fields of organic and inorganic synthesis,^8−10^ materials chemistry,^11^ and pharmaceutical sciences,^12,13^ we have therefore recently developed a new method that uses mechanochemistry for the modeling of oxidative degradation processes in APIs (Figure 1b).^14,15^ In general, several approaches were developed for such studies, including the use of H_2_O_2_, azoalkanes, radical initiators, or transition metals, covering several types of oxidations, i.e., one- or two-electron oxidation or free-radical oxidation.^16^ The mechanochemical oxidation of small organic molecules such as 5-hydroxymethyl furfural and lignin β-O-4 model compounds was reported before.^17,18^ Paudel and co-workers have recently applied mechanoactivation to evaluate the autoxidation propensity of amorphous drugs.^19^

Our previous work used^14^ Clopidogel hydrogen sulfate (**CLP**), a compound present in antiplatelet drugs, as a thienopyridine containing model compound. Different degradation products could be selectively obtained by ball-milling in short reaction times of less than 15 min. The nature of the product was found to be strongly dependent on the oxidant used. Specifically, *N*-oxidation of **CLP** to produce the *N*-oxide **CLP-DP-1** was the main reaction when using Oxone, the triple salt of potassium peroxymonosulfate, potassium hydrogen sulfate, and potassium sulfate (2KHSO_5_·KHSO_4_·K_2_SO_4_). With KNO_3_ and KMnO_4_, formation of the so-called *endo*-iminium species **CLP-DP-2** was the dominant process with reactions using these oxidants being generally much less selective, producing more ill-defined mixtures (Figure 1b).

To further evaluate whether the results of this model study are relevant for the degradation processes occurring in a commercially available drug containing this API, it is necessary to also perform forced degradation studies in the presence of the excipients that are present in commercial forms of **CLP**, such as Plavix (Figure 1c). To the best of our knowledge, such systematic investigations have not been done yet. In this contribution, we describe a systematic investigation of solid-state oxidation of **CLP** in the presence of excipients present in the commercially available drug Plavix, followed by mechanochemical degradation of the drug itself. The general applicability of our mechanochemical approach is further highlighted in a comparative analysis of related second generation P2Y12 antagonists,^20^ i.e., platelet aggregation inhibitors, thienopyridine-based drugs Ticlopidin-neuraxpharm and Efient, which contain Ticlopidine and Prasugrel as the APIs, respectively.

## Results and Discussion

### Drug-Excipient Stability Studies

**Scheme 1:**
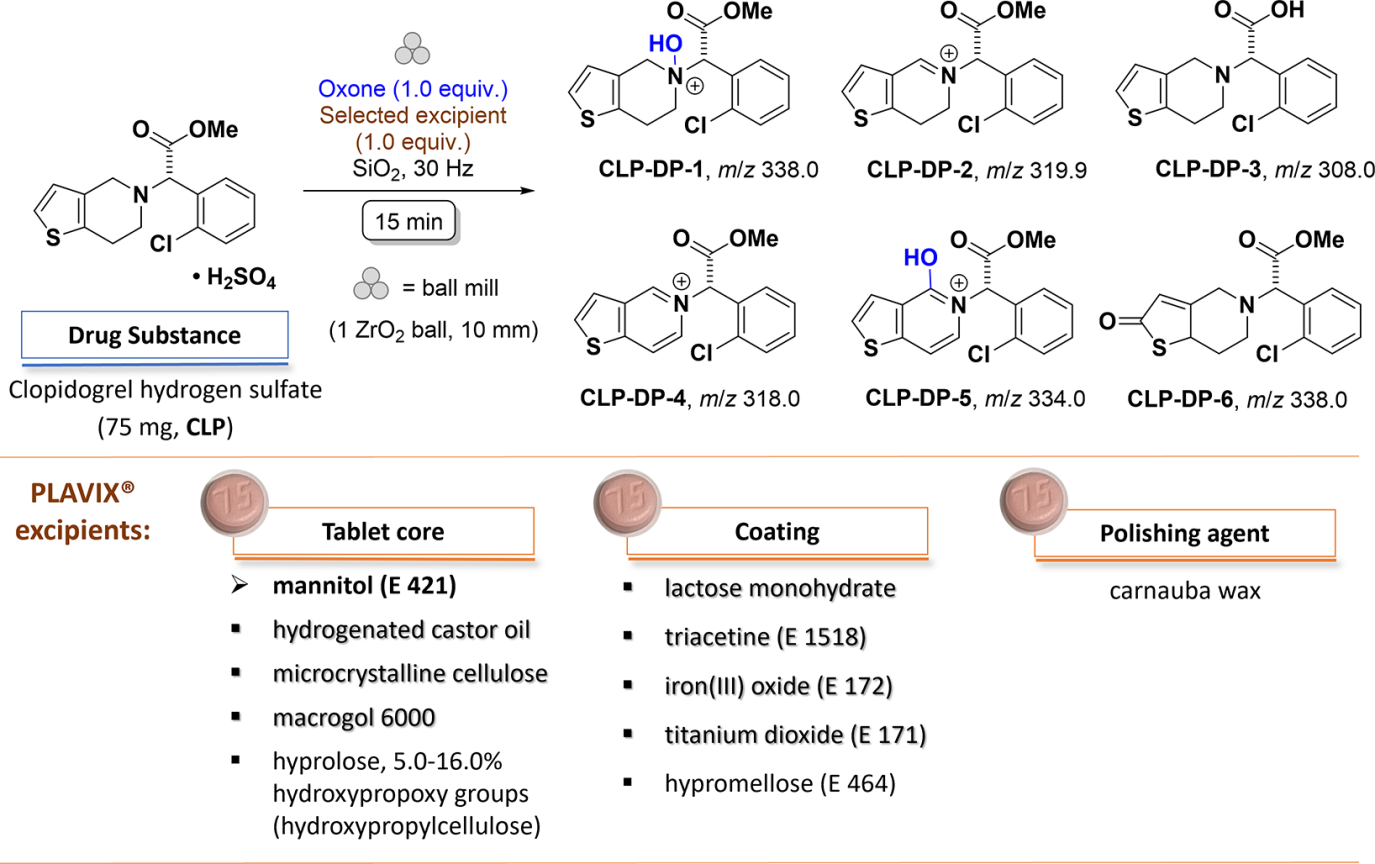
Top, Mechanochemical Oxidative Degradation of **CLP** in the Presence of Excipients (Bottom, One at a Time) and Main Degradation Products Detected by HPLC; Bottom, Summary of Excipients Present in Plavix The excipients marked with a text-shadow were investigated in this study. Mannitol (highlighted in bold) represents our excipient model.

The investigated form of Plavix contained 75 mg of **CLP** per tablet, as well as excipients listed in Scheme 1. To evaluate the influence of each of these compounds on the APIs degradation profile, we have first performed experiments in the presence of a single additive, i.e., each of the excipients in the formulation, one at a time. We have exclusively used Oxone for this study as this oxidant was found to give the most selective oxidation processes before. We furthermore wanted to compare solid-state data obtained here with previously described solution data.^21^ Following our previously described approach,^14^ we thus performed the mechanochemical studies using a commercially available mixer ball mill using ZrO_2_ jars and balls^22^ at a frequency of 30 Hz. Reaction mixtures contained **CLP**, the excipient of choice, oxone, and inert SiO_2_ as a grinding auxiliary that secures a minimum loading of the milling jars. Milling was done for reaction times of up to 15 min (Scheme 1), followed by workup using MeCN/water mixtures and LC-MS, NMR, and ATR-IR analysis. This approach resulted in **CLP** conversions of 30–50%. In line with previous data,^14^ longer reaction times resulted in higher degrees of degradation of **CLP**, producing less defined degradation profiles. Similarly, milling frequencies of less than 25 Hz gave no conversion of the API.

**Figure 2:**
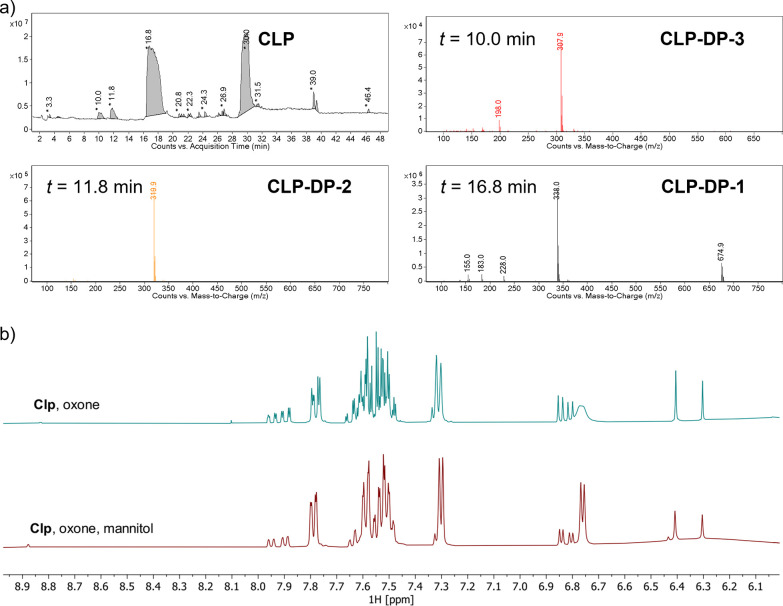
(a) LC-MS analysis of the main products of oxidative mechanochemical degradation of **CLP** with Oxone in the presence of mannitol along with MS data recorded at given retention times. (b) Comparison of ^1^H NMR spectra (CD_3_CN, 297 K) of the product of oxidative mechanochemical degradation of **CLP** with Oxone with and without mannitol. Both spectra are dominated by signals due to **CLP** and **CLP-DP-1**.

Excipients present in the core of the Plavix tablet are mannitol, cellulose, hydrogenated castor oil, macrogol 6000, and hydroxypropylcellulose. Starting with mannitol as an additive, we found that—similar to that as in previous studies—retention of the clopidogrel scaffold was observed, and **CLP-DP-1** was the dominant product (*m*/*z* 338.0), along with small amounts of the *endo*-iminium compound **CLP-DP-2** (*m*/*z* 319.9, Figure 2a), likely the product of water elimination from **CLP-DP-1**. ^1^H NMR spectroscopic analysis of the reaction mixtures produced data that were reported before for solid-state oxidative degradation of pure **CLP** using Oxone (Scheme 1, Figure 2b). The main product **CLP-DP-1** was present as a mixture of two diastereomers, confirmed by two sets of signals, including singlets due to the methine proton in the bridging position at 6.30 and 6.41 ppm as well as characteristic doublets for the thiophene protons at 6.80–6.85 and 7.90–7.95 ppm. These data confirm that the presence of mannitol does not affect the outcome of the degradation experiment. Similar sets of experiments were done using cellulose, hydrogenated castor oil, and macrogol 6000, and they all showed that the nature of the excipient did not affect the degradation profiles (Figures S2–S4). It should, however, be noted that partial hydrolysis of the ester group to produce **CLP-DP-3** was evident in reactions with cellulose and mannitol (*m*/*z* 307.9, Figure S1 and S2).

**Figure 3:**
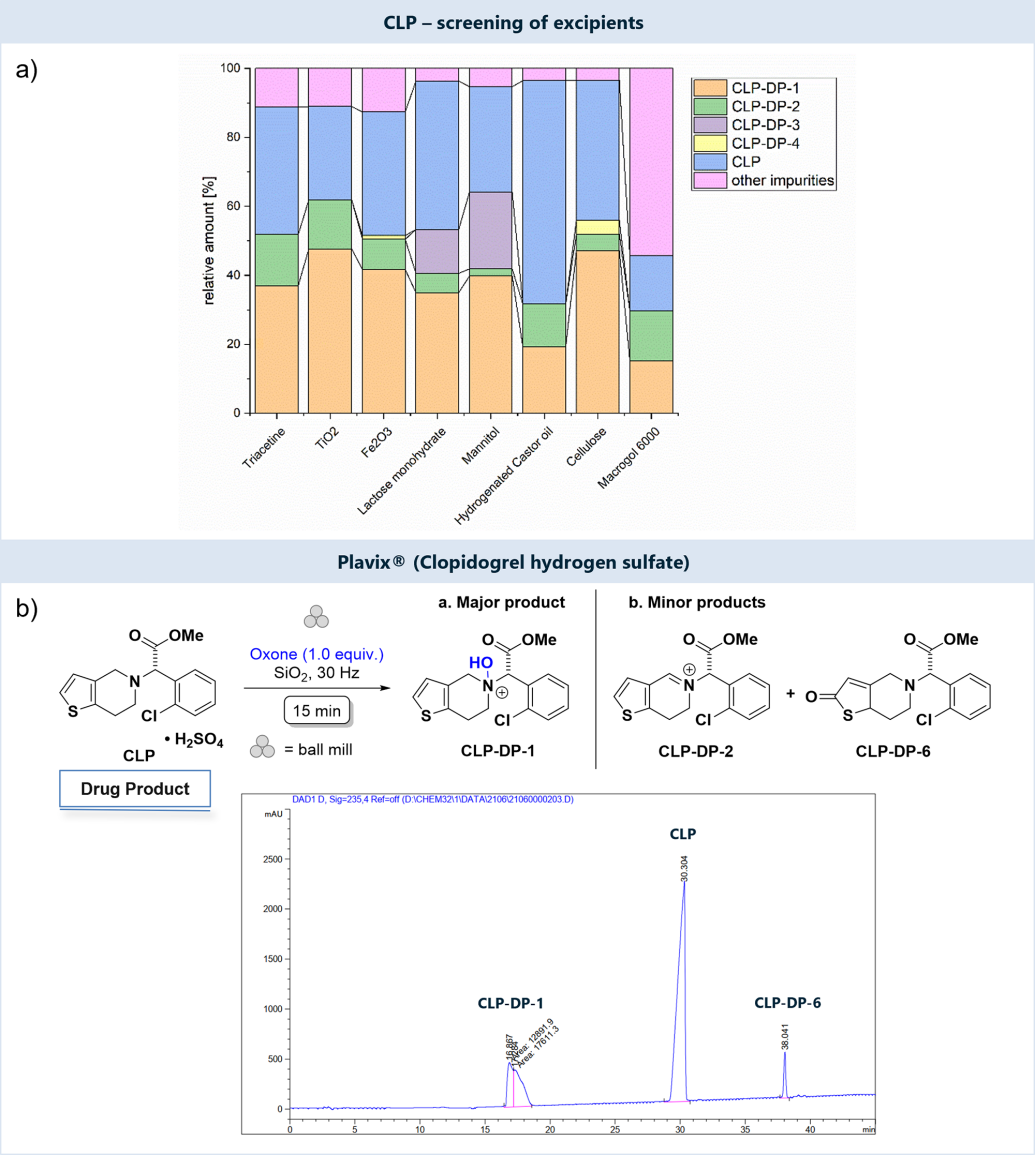
(a) Summary of LC-MS analysis of the products of oxidative mechanochemical degradation of **CLP** with Oxone and selected excipients (ZrO_2_ jars, 1 ball, 15 min, 30 Hz, in SiO_2_); (b) LC analysis of products of oxidative mechanochemical degradation of Plavix with Oxone.

Plavix further contains lactose monohydrate, hypromellose, triacetine (i.e., glycerol triacetate), Fe_2_O_3_, and TiO_2_ as part of the coating. Ball milling of **CLP** with any of these compounds in the presence of Oxone qualitatively produced the same degradation profiles as described before, containing **CLP-DP-1** and **CLP-DP-2** as the main species in all cases (Figure 3a).

Formation of the acid **CLP-DP-3** was detected for the addition of lactose monohydrate. A comparison of LC-MS traces obtained from reactions using different coating additives is shown in Figure S5–S8. Notably, peaks that could unequivocally be assigned to the *endo*-iminium compound **CLP-DP-2** showed significant tailing, an effect that was described before.^14^ Taken together, we conclude that neither the excipients present in Plavix nor the coating materials influence the nature of the products formed during oxidative degradation studies. It is therefore justified to use the entire tablet for further studies of mechanochemical oxidation. Indeed, milling of Plavix (tablet of 75 mg) with SiO_2_ and Oxone produced the same degradation profile as described above. The presence of the main oxidation products **CLP-DP-1** (44.2%) and **CLP-DP-2** (2.2%) along with unreacted **CLP** (41.9%) could be confirmed by LC-MS (Figures 3b, S10, and S11) and detected by NMR analysis (Figure S18). Furthermore, also the ratio of these main degradants remained the same in all experiments (Figure 3b), indicating that our approach can be used for the predictive forced degradation of finished pharmaceutical products. Based on this finding, we have next extended our studies to related platelet inhibitor drugs containing APIs with thienopyridine motifs. The chosen drugs were Ticlopidin-neuraxpharm (ticlopidine hydrochloride, **TIC**, 250 mg) and Efient (prasugrel hydrochloride, **PRA**, 10 mg), and their oxidative degradation behavior in the presence of Oxone was tested.

### Stability of Related Thienopyridine Drugs

#### Ticlopidin-neuraxpharm

**Figure 4:**
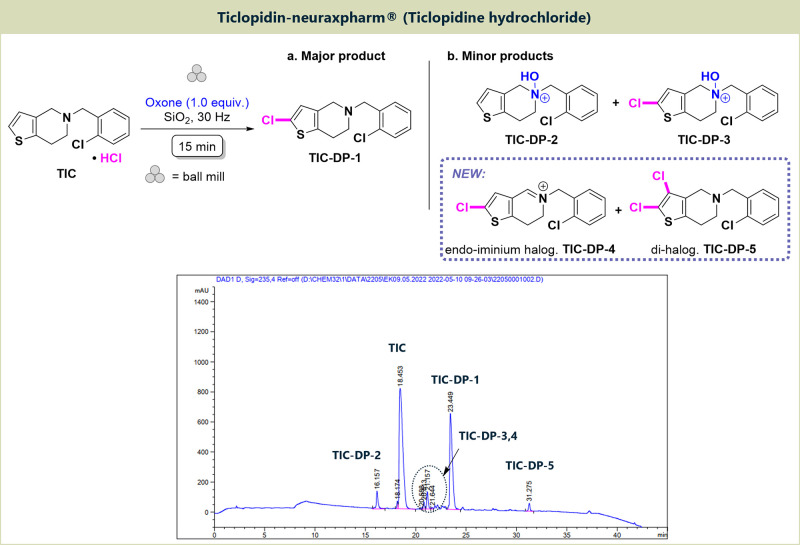
Mechanochemical oxidative degradation of Ticlopidin-neuraxpharm, containing **TIC** and main degradation products detected by HPLC.

The stability of different ticlopidine formulations under the International Council for Harmonisation (ICH) accelerated test conditions (40 °C/75% relative humidity [RH], 3 and 6 months^23^) was studied in the past.^24^ To the best of our knowledge data for the stability in the solid state under oxidative conditions have not been reported yet. Ticlopidin-neuraxpharm contains 250 mg of **TIC** as the API. Ball milling of half a tablet with Oxone using the above-mentioned reaction conditions for 15 min, followed by aqueous workup, produced three previously reported degradation products **TIC-DP-1**, **TIC-DP-2**, and **TIC-DP-3**. In these products, *N*-hydroxylation and chlorination of the thiophene moiety occurred, the latter functionalization arising from the chloride counteranion present in the drug (Figure 4). In addition, two new degradation products could be identified based on LC-MS, showing *m*/*z* values of 296 and 330. We assigned these to an iminium species that was chlorinated in 2-position at the thiophene unit (**TIC-DP-4**, exact mass for C_14_H_12_Cl_2_NS^+^ 296.0062) as well as a structure that is dichlorinated in 2,3-position of the thiophene ring (**TIC-DP-5**, exact mass for C_14_H_12_Cl_2_NS^+^ 330.9756). The formation of these degradation products is in line with previous studies of oxidative **CLP**(25) and **TIC**(26−29) degradation in solution, where oxidative 2-halogenation of the thiophene moiety was a dominant reaction. Attempts to further analyze these products by NMR spectroscopy failed (Figures S19 and S20) as we were unable to separate and isolate any of the species shown in Figure 4.

#### Efient

**Figure 5:**
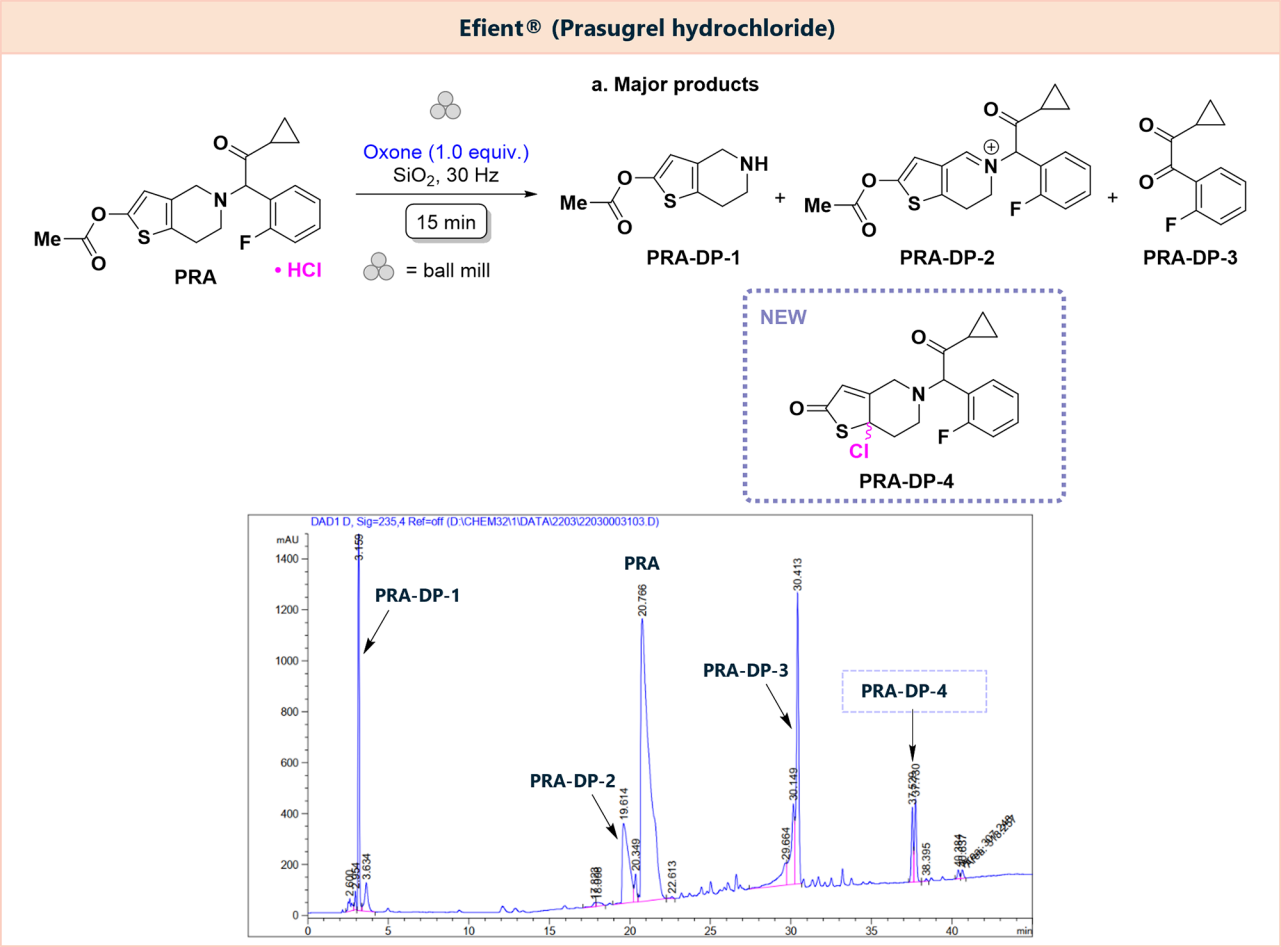
Mechanochemical oxidative degradation of Efient, containing **PRA** and main degradation products detected by HPLC.

Efient is a highly active platelet inhibitor drug, containing 10 mg of **PRA** as API. Its impurities have been identified, and the degradation behavior in solution has been studied by several groups in the past.^30−33^ An extensive study by Jansen and co-workers^33^ includes the solid-state stress testing of **PRA** upon prolonged exposure to elevated temperature at high and low relative humidity (RH), also under photochemical conditions. While the compound is stable under the stress conditions of heat and light at low humidity, heating and an increase in humidity (60 °C/75% RH) are reported to result in approximately 27% degradation after 28 days with the main products showing either oxidation at the thiophene moiety or hydrolytic cleavage of the oxidation products. Oxidative degradation in solution was studied using a variety of different oxidants, producing a wide range of new degradation products, derived from simple oxidation of the thienopyridine fragment (e.g., **PRA-DP-2**, Figure 5), subsequent cleavage of the API structure (e.g., **PRA-DP1** and **PRA-DP-3**), or selective hydrolysis of the acetyl-thiophene fragment.^33^

Forced oxidative degradation of **PRA** in a formulation was tested using our established procedure using three tablets of Efient. After a milling time of 15 min, four main products could be identified by HPLC (Figure 5) and LC-MS (Figures S14–S16).

*endo*-Iminium compound **PRA-DP-2** (*m*/*z* 372) was formed, likely due to initial *N*-hydroxylation, followed by elimination of water. Additional products include the diketone **PRA-DP-3** (*m*/*z* 192) and an acetylated thienopyridine **PRA-DP-1** (*m*/*z* 198), as well as a product that was formed by chlorination of the deacylated/oxidized **PRA** scaffold (**PRA-DP-4**, *m*/*z* 366). While hydroxylation in the 7-position of the thienopyridine moiety was reported before under acidic conditions, incorporation of the halide counteranion was not reported to date. LC-ESI-HRMS analysis (Figure S16, Table S5) of this mixture confirms these assignments and reveals the formation of further degradation products by displacement of the fluoroarene (*m*/*z* 240.0699) or oxidation of the thiophene unit (*m*/*z* 332.1132, 330.0972, 348.1079). Species generated by hydroxylation of the *N*-heterocycle (*m*/*z* 388.1028) as well as cleavage of the cyclopropyl ketone moiety (*m*/*z* 320.0761) were further observed.

**Figure 6:**
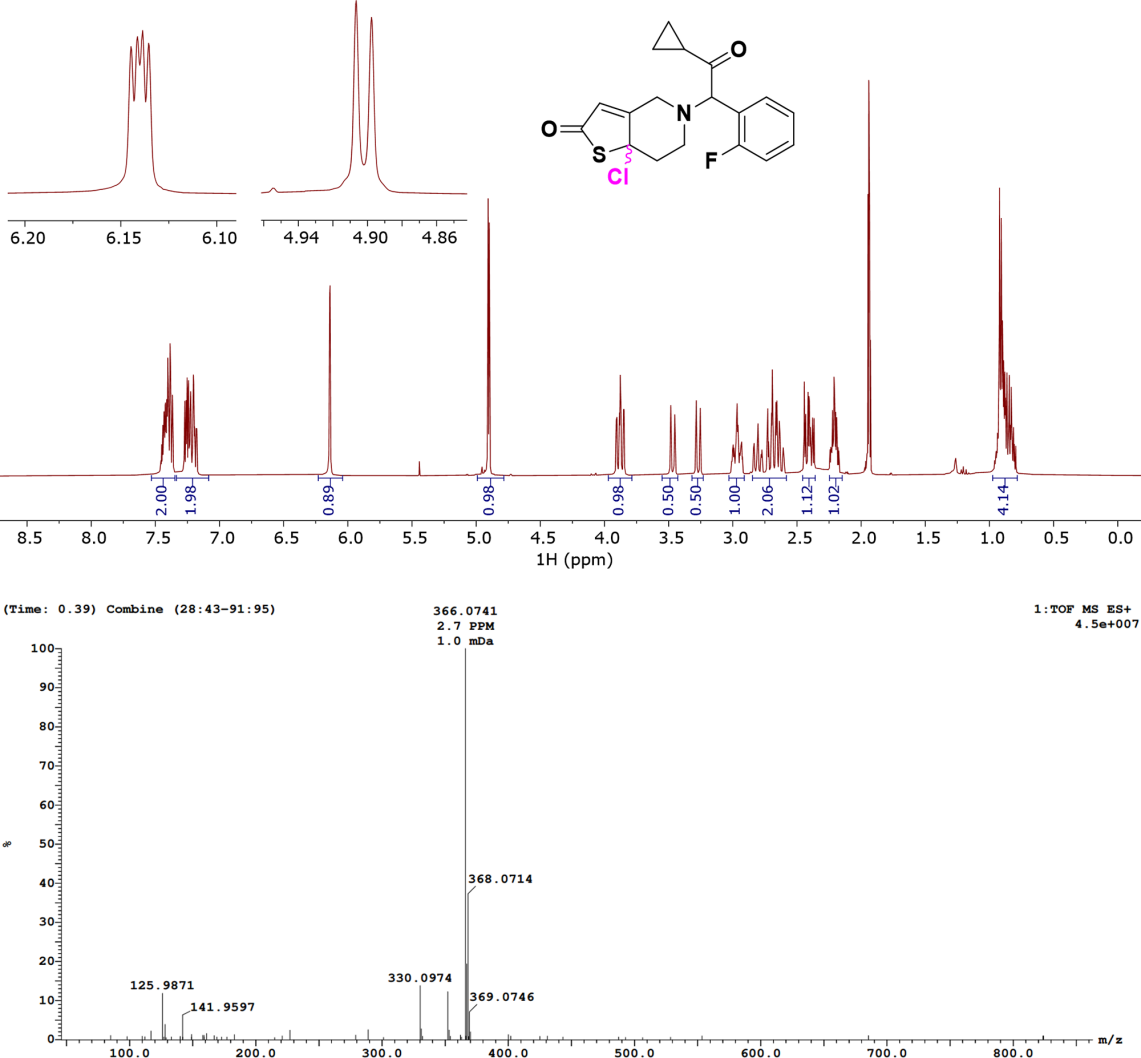
Top: ^1^H NMR spectrum of halogenated product **PRA-DP-4** (CD_3_CN, 400 MHz). The inset shows the characteristic set of signals for two diasteromers (thiophene CH proton, 6.14 ppm; CH proton vicinal to N, 4.90 ppm). Bottom: HRMS data of halogenated product **PRA-DP-4** (C_18_H_17_ClNO_2_FS, calcd [M]^+^, 366.0731 found, 366.0741; 2.7 ppm error).

We have next prepared the previously unknown compound **PRA-DP-4** by mechanochemical chlorination of **PRA** and isolated the previously unknown compound in 77% purity. Analysis by high-resolution mass spectrometry (calculated for [C_18_H_17_ClNO_2_FS]^+^, 366.0731; found, 366.0741; 2.7 ppm error, Figure 6) and multinuclear 1D and 2D NMR spectroscopy (Figures S24–S29) confirm the structural assignment as a singly chlorinated derivative. **PRA-DP-4** is formed as a mixture of two diastereomers, showing characteristic overlapping doublet resonances for the remaining CH proton at the thiophenone unit (6.14 ppm) and the bridging methine proton at 4.90 ppm. In the ^19^F NMR spectrum, the compound shows multiplet signals at −116.65 and −116.44 ppm. This result of chloride incorporation is reminiscent of previous studies of oxidative halogenation of **TIC** and **CLP** in solution. In these cases, introduction of the halogen atom occurs in the 2-position of the thiophene unit. As this position is already oxidized in case of **PRA**, the 7-position is activated, and chlorination takes place at this site.

In general, all thienopyridines discussed in this study show similar degradation profiles. As discussed before,^11,14^ hydroxylation of the *N*-heterocycle is common for forced oxidative degradation of **CLP** and **TIC**. Formation of *endo*-iminium species occurs for all APIs, as the carbon atom bridging the thiophene and the nitrogen atom is highly susceptible to oxidation. Activation of the halogenated arene unit is not observed. For **TIC** and **PRA**, oxidative incorporation of the chloride counteranion into the drug scaffold was found. This type of reaction was reported by Krake and Baumann before and can be used for the selective halogenation of thienopyridine using simple metal halides.^25,26^ Whereas this occurs in 2- and 3-position of the thiophene for **TIC**, the Cl is added in 7-position of the thienopyridine unit for **PRA**. In general, **PRA** as the higher functionalized compound shows a much more complex degradation profile with four main degradants. Known products **PRA-DP-1**, **PRA-DP-2**, and **PRA-DP-3** obtained from forced oxidative degradation of the **PRA** containing drug Efient in the solid-state resemble those reported before in solution studies.^33^ However, mechanochemical oxidative degradation produces a much less complex degradation profile compared to most oxidation reactions performed in solution. The formation, characterization and isolation of a chlorinated oxidation **PRA-DP-4** product was not reported to date and shows the power of our highly efficient mechanochemical approach.

## Conclusion

In summary, we have presented the mechanochemical forced oxidative degradation of three structurally related thienopyridine containing antiplatelet drugs. Model studies using clopidogrel hydrogensulfate, in combination with selected excipients, showed that the excipient did not affect the nature of the products formed during oxidative degradation with Oxone. The results obtained from forced degradation studies with either the isolated API, the API/excipient mixture, or the drug product were thus very similar. We therefore conclude that reproducible and meaningful degradation profiles can be obtained for thienopyridine drugs in short reaction times using only the API. Correlations between the degradation profiles formed using this approach and long-term stability of drug products will be assessed in future studies. The mixture of degradation products produced via mechanochemistry might be considered for the development of a stability-indicating HPLC method beside the classical forced degradation. Furthermore, this study reveals the importance of selecting a suitable salt form for drugs during the preformulation phase of drug development. Under ball milling conditions, halide counterions can participate in oxidative solid-state degradation processes and lead to products that are not observable with other counterions.

In general, mechanochemical studies of transformations of small organic molecules, in particular of pharmaceuticals,^13^ are typically done, aiming at the *synthesis* of certain structural motifs. Our work highlights the potential of this approach for the targeted *degradation* of specific structural motifs that could not only be relevant for the pharmaceutical sciences as discussed here, but also for organic synthesis in general. In future work, we will attempt to transfer this mechanochemical approach to other drug families and evaluate the role of other stimuli such as light or temperature for the forced degradation process. Our approach has the potential to significantly simplify the acquisition of stability data and degradation profiles that is required for approval of new drugs.

## Methods

Unless otherwise indicated, all commercially available starting materials and solvents were purchased and used as received without further purification. Plavix (Sanofi-Aventis GmbH, Austria; SN 100D2NCF06AH9W), Ticlopidin-neuraxpharm (Neuraxpharm Arzneimittel GmbH, Germany; SN FGGDWWWHLX1GWG), and Efient (Daiichi Sankyo Austria GmbH, Austria; SN 1000005010639497) were used as received. Ball milling experiments were carried out with a Retsch MM400 (Retsch GmbH, Retsch-Allee 1–5, 42781 Haan, Deutschland) ball mill. ZrO_2_–Y (zirconia dioxide stabilized with Yttria) milling jars (10 mL) and one ZrO_2_–Y milling ball (10 mm) were used as milling equipment.

### API-Excipient Stability Studies

The forced solid state oxidative degradation studies were done by ball milling mixtures of the clopidogrel hydrogen sulfate (**CLP**), the excipients and Oxone in a mixer mill. For this, 100 mg of **CLP**, 73 mg of Oxone (1.0 equiv), 250 mg of SiO_2_ and one ZrO_2_–Y ball (*d* = 10 mm) were placed in a ZrO_2_–Y jar, and one equivalent of the respective excipient was added (86 mg for lactose monohydrate, 29 mg for TiO_2_, 52 mg for triacetine, 78 mg for cellulose, 88 mg for mannitol, 38 mg for Fe_2_O_3_, 100 mg for Hydrog. Castor Oil, and 477 mg for Macrogol 6000, respectively). The jar was closed, transferred to the ball mill, and the mixture was allowed to react at a frequency of 30 Hz for 15 min. The jar was opened and thoroughly rinsed with approximately 10–15 mL of MeCN, followed by filtration through a paper filter. The filtrate was concentrated to dryness *in vacuum* to produce oily mixtures. For HPLC analysis, 1 mg/mL **CLP** equivalents were weighed directly and dissolved in mobile phase (Hex/EtOAc, 4:1).

### Drug Salt Stability Studies of Thienopyridine Drugs

The procedure is identical with the above using the following amounts of compounds:

(a) Plavix, 75 mg (we used 1 pill, **CLP**), 55 mg of Oxone (1.0 equiv), and 250 mg of SiO_2_.
(b) Ticlopidin-neuraxpharm, 250 mg (we used half a pill = 125 mg, **TIC**), 256 mg of Oxone (1.0 equiv), and 250 mg of SiO_2_.
(c) Efient, 10 mg (we used 3 pills = 30 mg, **PRA**), 45 mg of Oxone (1.0 equiv), and 250 mg of SiO_2_.

All the reactants were placed in a ZrO_2_–Y jar with one ZrO_2_–Y ball (*d* = 10 mm).

### Synthesis of PRA-DP-4

**PRA** (API; free base, 300 mg), NaCl (1.5 equiv., 70 mg), Oxone (1.0 equiv, 493 mg), and SiO_2_ (250 mg) were placed in a milling jar and milled for 15 min at a frequency of 30 Hz. After this period, the crude solid material was purified using column chromatographic workup to provide a dark orange/brown oil (50 mg, 17.0%) (Hex/EtOAc, 4:1). *R*_f_: 0.72 (Hex/EtOAc, 4:1).

## Supporting Information Available

Experimental details, full HPLC and LC-MS data, and characterization data for **PRA-DP-4** (PDF)
