# Supplementary material for: Mechanochemical Oxidative Degradation of Thienopyridine Containing Drugs: Toward a Simple Tool for the Prediction of Drug Stability
Source: ACS Cent Sci. 2023 May 16;9(6):1150–9. doi: 10.1021/acscentsci.3c00167 (PMC10311657; doi:10.1021/acscentsci.3c00167)
Supplement: Supplementary file 1 — oc3c00167_si_001.pdf [file oc3c00167_si_001.pdf]

Supporting Information

Mechanochemical oxidative degradation of thienopyridine containing drugs: Toward a simple tool for the prediction of drug stability

Everaldo F. Krake[a], Laura Backer[b], Benjamin Andres[a], Wolfgang Baumann[a], Norbert Handler[c], Helmut Buschmann[c], Ulrike Holzgrabe*[b], Carsten Bolm*[d], Torsten Beweries[a]*

[a] Leibniz-Institut für Katalyse e.V., Albert-Einstein-Str. 29a, 18059 Rostock, Germany. torsten.beweries@catalysis.de

[b] Universität Würzburg, Institut für Pharmazie und Lebensmittelchemie, Am Hubland, 97074 Würzburg, Germany. ulrike.holzgrabe@uni-wuerzburg.de

[c] RD&C Research, Development & Consulting GmbH, Neuwaldegger Strasse 35/2/3, 1170 Vienna, Austria

[d] RWTH Aachen University, Institut für Organische Chemie, Landoltweg 1, 52074 Aachen, Germany. carsten.Bolm@oc.rwth-aachen.de

Table of contents

General information S2
HPLC and LC-MS data S4
NMR data S28
HRMS data of PRA-DP-4 S36
References S37

General Information

Unless otherwise indicated, all commercially available starting materials and solvents were purchased and used as received without further purification. Plavix® (Sanofi-Aventis GmbH, Austria; SN 100D2NCF06AH9W), Ticlopidin neuraxpharm® (Neuraxpharm Arzneimittel GmbH, Germany; SN FGGDWWWHLX1GWG), and Efient® (Daiichi Sankyo Austria GmbH, Austria; SN 1000005010639497) were used as received.

Ball milling experiments were carried out with a Retsch MM400 (Retsch GmbH, Retsch-Allee 1-5, 42781 Haan, Deutschland) ball mill. ZrO_2-Y (zirconia dioxide stabilized with Yttria) milling jars (10 mL) and one ZrO_2-Y milling ball (10 mm) were used as milling equipment.

HPLC measurements were conducted with an Agilent Technologies instrument (1100 series). The detection wavelength was λ = 235 nm, and all information are given in Table S1.

Table S1. Summary of LC methods.

	HPLC	LC for MS
Column	Zorbax Eclipse Plus C8 column (double-endcapped, carbon load 7%, 250 × 4.6 mm, 5 µm particles)	Zorbax Eclipse Plus C_8, 250 x 4.6 mm, 5 µm particle size
Mobile phase	A: H_2O + 0.1% formic acid (v/v)<br>B: ACN + 0.1% formic acid (v/v)	A: H_2O + 2% ACN + 0.1% formic acid (v/v)<br>B: ACN + 2% H_2O + 0.1% formic acid (v/v)
Elution mode [% of B in v/v]	0 - 10 min: 20% B,<br>10 - 40 min: 20→80% B,<br>40 - 45 min: 80% B,<br>45 - 48 min: 80→20% B,<br>48 - 50 min: 20% B	0 - 2 min: 25%<br>2 - 20 min: 25→75%<br>20 - 25 min: 75%<br>25 - 27 min: 75→25%<br>27 - 30 min: 25%
Flow rate	1.0 mL/min	1.0 mL/min
Sample concentration	1 mg mL^{-1} (ACN)	1:5 (v/v) in ACN
Injection volume	15 µL	2 µL
Temperature	25 °C	40 °C

High-resolutions mass spectra (HRMS, ESI+ mode) measurements were performed on an Agilent 6210 instrument. Chromatography for HRMS measurements was performed on an Agilent Infinity II system coupled to a Sciex X500R QTOF mass spectrometer equipped with a Turbo V™ Ion Source (ESI). Further information are given in Table S1.

Table S2. Summary of MS methods.

TOF MS	ESI+
Accumulation time [s]	0.25
Collision energy ± spread [V]	10 ± 0
**IDA**	
Accumulation time [s]	0.1
Collision energy± spread [V]	35 ± 15
Maximum candidate ions	10
Intensity threshold [counts/s]	10
Dynamic background subtraction	True

Parameter	ESI+
Gas 1	50
Gas 2	50
Temperature [°C]	450
Spray Voltage [V]	5500
Mass range	50-1000
Curtain gas	25
CAD gas	7
Declustering Potential ± spread [V]	70 ± 10

NMR spectra were recorded on a Bruker AVANCE III HD 400 MHz spectrometer and recorded at 297 K in CD_3CN as the solvent. Chemical shifts are reported in ppm (δ) and were referenced to residual CH_3CN (δ_H = 1.94 ppm, δ_C = 1.32 ppm). 20 mg of the respective compound were dissolved in 0.4 mL of CD_3CN and were used for ^1H, ^{13}C, ^{19}F NMR, ^1H-^1H COSY, ^1H-^{13}C HSQC and ^1H-^{13}C HMBC analysis.

Recording of one-dimensional NMR spectra

The pulse conditions were as follows: **^1H NMR**, spectra (pulse sequence = *zg30*): number of data points (TD) = 43008, number of scans (NS) = 32, dummy scans (DS) = 2, spectra width (SWH) = 8012.820 Hz, acquisition time (AQ) = 2.6837 sec, spectrometer operating frequency (SFO1) = 400.13 MHz, π/2 pulse for ^1H (P1) = 14.30 µs, relaxation delay (D1) = 1.27 s, line broadening (LB) = 0.10 Hz. **^{13}C NMR** spectra (pulse sequence = *zgpg30*): TD = 43702, NS = 512, DS = 2, SWH = 29411.766 Hz, AQ = 0.7429 sec, SFO1 = 100.626 MHz, LB = 1.00 Hz, D1 = 2.0 sec, P1 = 10.0 µs. **^{19}F NMR** spectra (pulse sequence = *zgflqn*): TD = 131072, NS = 16, DS = 4, SWH = 89285.711 Hz, AQ = 0.734 sec, SFO1 = 376.46 MHz, LB = 0.30 Hz, D1 = 1.0 sec, P1 = 22.0 µs.

Recording of two-dimensional NMR spectra

^1H-^1H COSY (pulse sequence = *cosygpppqf*): TD = 2048 (F2), TD = 147 (F1) NS = 2, DS = 16, SFO1 = 400.132 MHz, LB = 0 Hz, D1 = 2.00 sec. **HSQC** (pulse sequence = *hsqcedetgp*): TD = 1024 (F2), TD = 256 (F1) NS = 2, DS = 16, SFO1 = 400.132 (F2) MHz, SFO1 = 100.622 (F1) MHz, LB = 0 Hz, D1 = 1.50 sec. **HMBC** (pulse sequence = *hmbcgpndqf*): TD = 2048 (F2), TD = 256 (F1) NS = 8, DS = 16, SFO1 = 400.132 (F2) MHz, SFO1 = 100.622 (F1) MHz, LB = 0 Hz, D1 = 2.0 sec.

HPLC and LC-MS data

*Mechanical oxidative degradation of Clopidogrel hydrogen sulfate (**CLP**, drug substance) + excipients*

**Instrument Name**	LCMS	**Data Filename**	D:\Chem32\1\Data\2105\21050606.D
**Acq Method**	Andres - SCAN Pos_m	**Sample Name**	PS 125 OXONE
**DA Method**	HRMS.m	**Position**	Vial 43
**User Name**	SYSTEM	**Comment**	ACN+0,1%HCOOH/H2O+0.1%HCOOH Gradient

User Chromatograms

Fragmentor Voltage 0 **Collision Energy** 0 **Ionization Mode** ESI

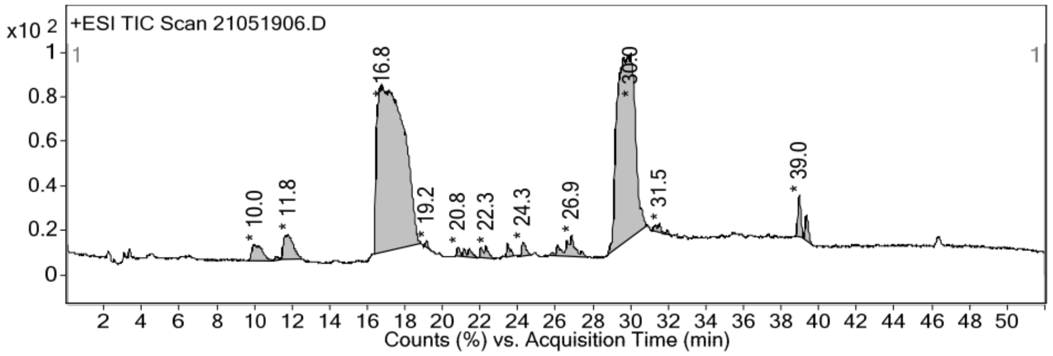

Integration Peak List

Peak	Start	RT	End	Height	Area	Area %
1	9.6	10	11	1435317	55952891	3.81
2	11.5	11.8	12.5	2272543	76880801	5.24
3	16.4	16.8	18.8	15025721	1467951532	100
4	21.9	22.3	22.8	1023200	25106522	1.71
5	23.3	23.5	23.8	1157958	14094255	0.96
6	24.1	24.3	24.7	1254055	18496750	1.26
7	25.7	26.9	27.7	1854695	70517524	4.8
8	28.8	30	30.8	16602444	1129745508	76.96
9	38.7	39	39.1	3672228	41137839	2.8
10	39.1	39.4	39.7	2311542	26456970	1.8

Figure S1. LC-MS data of mixture obtained after mechanochemical reaction of **CLP**, mannitol and Oxone®.

**Instrument Name**	LCMS	**Data Filename**	D:\Chem32\1\Data\2105\21050604.D
**Acq Method**	Andres - SCAN Pos_m	**Sample Name**	PS 124 OXONE
**DA Method**	HRMS.m	**Position**	Vial 43
**User Name**	SYSTEM	**Comment**	ACN+0,1%HCOOH/H2O+0.1%HCOOH Gradient

User Chromatograms

Fragmentor Voltage 0 **Collision Energy** 0 **Ionization Mode** ESI

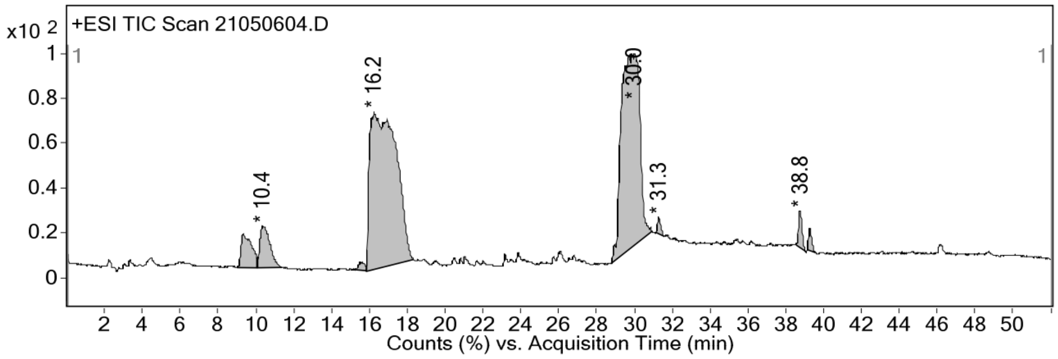

Integration Peak List

Peak	Start	RT	End	Height	Area	Area %
1	9	9.3	10.1	2970665	115716020	8.6
2	10.1	10.4	11.4	3667078	137119162	10.19
3	15.2	15.6	15.8	704588	16511075	1.23
4	15.8	16.2	18.3	13948596	1345252978	100
5	28.7	30	30.9	16922717	1158307302	86.1
6	31.1	31.3	31.6	1468532	18851131	1.4
7	38.5	38.8	39.1	3284840	41324268	3.07
8	39.1	39.3	39.6	1936737	21859009	1.62

User Spectra

Figure S2. LC-MS data of mixture obtained after mechanochemical reaction of **CLP**, cellulose and Oxone®.

**Instrument Name**	LCMS	**Data Filename**	D:\Chem32\1\Data\2203\220325--008.D
**Acq Method**	Andres - SCAN Pos_m	**Sample Name**	PS152 OXONE
**DA Method**	HRMS.m	**Position**	Vial 47
**User Name**	SYSTEM	**Comment**	Methode Andres

User Chromatograms

Fragmentor Voltage 0 **Collision Energy** 0 **Ionization Mode** ESI

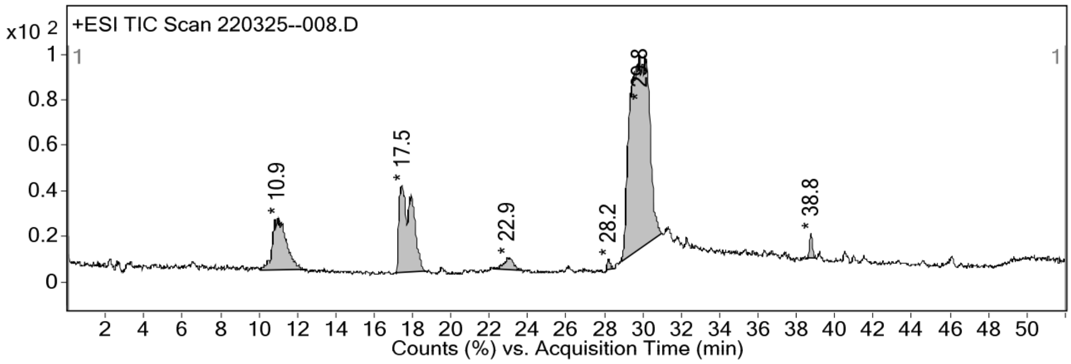

Integration Peak List

Peak	Start	RT	End	Height	Area	Area %
1	10	10.9	12.3	1822605	93439860	19.16
2	17.1	17.5	18.7	3061014	144341105	29.6
3	22.2	22.9	23.7	415004	14066019	2.88
4	28.1	28.2	28.5	354573	4065463	0.83
5	28.9	29.8	31	6819399	487635636	100
6	38.6	38.8	39	857476	8513166	1.75

User Spectra

Figure S3. LC-MS data of mixture obtained after mechanochemical reaction of **CLP**, hydrogenated castor oil and Oxone®.

**Instrument Name**	LCMS	**Data Filename**	D:\Chem32\1\Data\2203\220325--004.D
**Acq Method**	Andres - SCAN Pos_m	**Sample Name**	PS151 OXONE
**DA Method**	HRMS.m	**Position**	Vial 43
**User Name**	SYSTEM	**Comment**	Methode Andres

User Chromatograms

Fragmentor Voltage 0 **Collision Energy** 0 **Ionization Mode** ESI

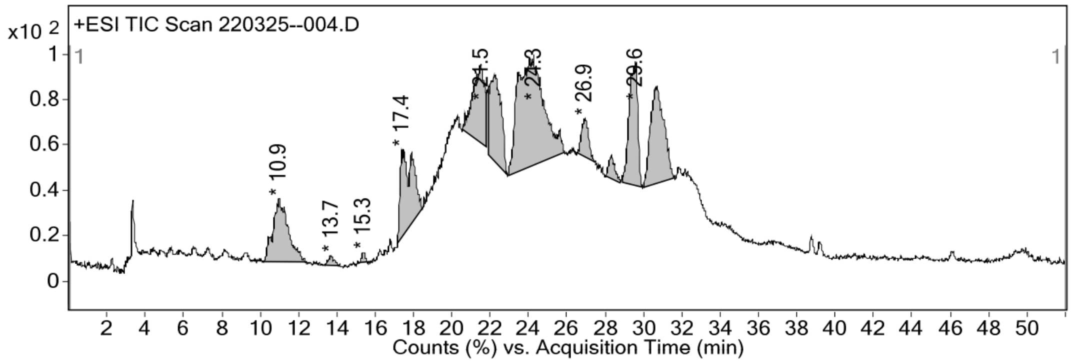

Integration Peak List

Peak	Start	RT	End	Height	Area	Area %
1	10.1	10.9	12.4	1964339	110191532	34.17
2	13.3	13.7	14.1	308494	6399000	1.98
3	17.1	17.4	18.5	2736644	115464130	35.81
4	20.5	21.5	21.8	2451277	102259474	31.71
5	21.9	22.2	22.8	2678975	111868125	34.69
6	22.9	24.3	25.9	3473443	322447099	100
7	26.5	26.9	27.5	1172428	29311620	9.09
8	27.9	28.3	28.8	713707	15383848	4.77
9	28.9	29.6	29.9	3897660	122328230	37.94
10	30	30.7	31.6	3024298	148592436	46.08

Figure S4. LC-MS data of mixture obtained after mechanochemical reaction of **CLP**, Macrogol 6000 and Oxone®.

**Instrument Name**	LCMS	**Data Filename**	D:\Chem32\1\Data\2101\21012003.D
**Acq Method**	Andres - SCAN Pos_m	**Sample Name**	PS120-10min
**DA Method**	HRMS.m	**Position**	Vial 43
**User Name**	SYSTEM	**Comment**	ACN+0,1%HCOOH/0,1%HCOOH in H2O Gradient

User Chromatograms

Fragmentor Voltage 0 **Collision Energy** 0 **Ionization Mode** ESI

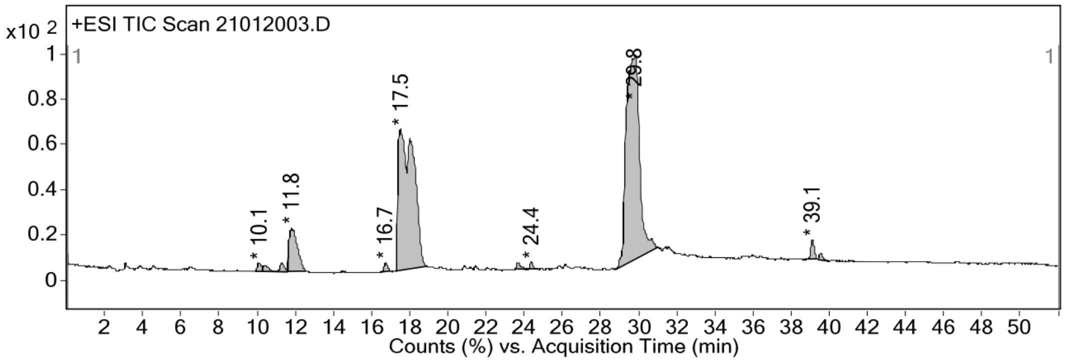

Integration Peak List

Peak	Start	RT	End	Height	Area	Area %
1	9.9	10.1	10.3	874473	12640783	1.24
2	10.3	11.3	11.6	874449	27624426	2.71
3	11.6	11.8	12.5	4455268	132729042	13.02
4	16.5	16.7	17	927753	11143167	1.09
5	17.2	17.5	18.9	14473575	828158936	81.26
6	23.5	23.7	24.1	630533	9623077	0.94
7	24.2	24.4	24.7	702079	6573051	0.64
8	28.8	29.8	31	21005071	1019106274	100
9	38.8	39.1	39.3	1939516	22924215	2.25
10	39.4	39.6	40	731469	8978113	0.88

Figure S5. LC-MS data of mixture obtained after mechanochemical reaction of **CLP**, lactose and Oxone®.

**Instrument Name**	LCMS	**Data Filename**	D:\Chem32\1\Data\2103\21040905.D
**Acq Method**	Andres - SCAN Pos_m	**Sample Name**	PS 123 Oxone
**DA Method**	HRMS.m	**Position**	Vial 44
**User Name**	SYSTEM	**Comment**	ACN+0,1%HCOOH/H2O+0.1%HCOOH Gradient

User Chromatograms

Fragmentor Voltage 0 **Collision Energy** 0 **Ionization Mode** ESI

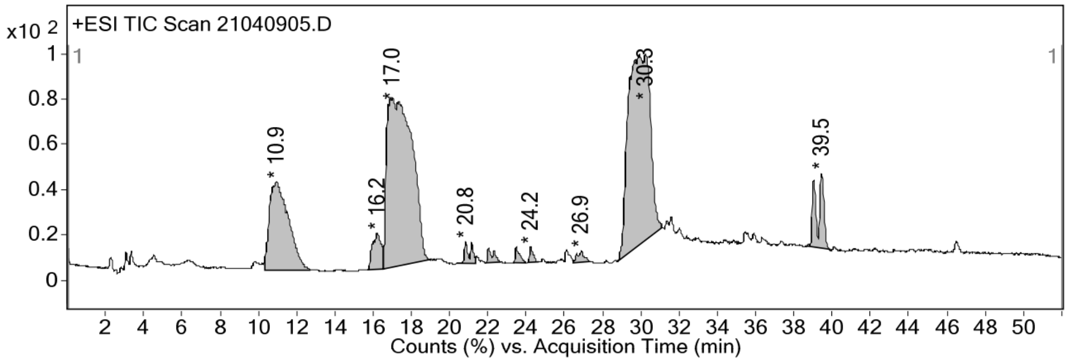

Integration Peak List

Peak	Start	RT	End	Height	Area	Area %
1	10.3	10.9	12.7	8141337	569577253	39.91
2	15.7	16.2	16.5	3314984	107448842	7.53
3	16.5	17	19	15600616	1425913338	99.92
4	20.4	20.8	21.4	1976121	41710206	2.92
5	21.8	22	22.7	1374142	28109311	1.97
6	23.3	23.5	24	1423074	21891307	1.53
7	24.1	24.2	24.7	1413484	17813548	1.25
8	26.5	26.9	27.4	1037330	25055185	1.76
9	28.8	30.3	31.1	17127208	1427060095	100
10	38.7	39.5	39.9	6903801	187141961	13.11

Figure S6. LC-MS data of mixture obtained after mechanochemical reaction of **CLP**, triacetine and Oxone®.

Instrument Name LCMS | **Data Filename** D:\Chem32\1\Data\2105\21052104.D
Acq Method Andres - SCAN Pos_m | **Sample Name** PS 126 OXONE
DA Method HRMS.m | **Position** Vial 43
User Name SYSTEM | **Comment** ACN+0,1%HCOOH/H2O+0.1%HCOOH Gradient

User Chromatograms

Fragmentor Voltage 0 **Collision Energy** 0 **Ionization Mode** ESI

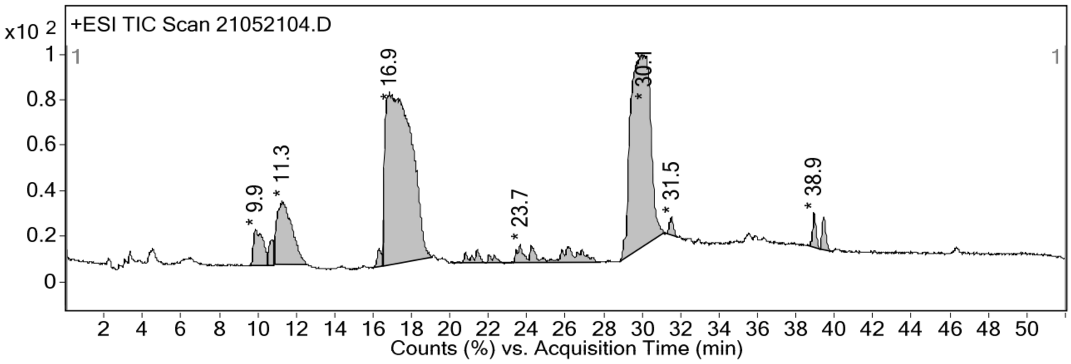

Integration Peak List

Peak	Start	RT	End	Height	Area	Area %
1	9.5	9.9	10.5	3015274	102413310	7.61
2	10.5	10.7	10.8	2149801	34980831	2.6
3	10.9	11.3	12.6	5250189	283560915	21.08
4	15.9	16.3	16.5	1472164	23095769	1.72
5	16.5	16.9	19	14211652	1345411624	100
6	20.1	23.7	27.9	1514938	192203946	14.29
7	28.8	30.1	31.1	15733216	1155005151	85.85
8	31.2	31.5	31.9	1494452	19925546	1.48
9	38.6	38.9	39.3	2829683	34969108	2.6
10	39.3	39.4	39.9	2745683	36216322	2.69

Figure S7. LC-MS data of mixture obtained after mechanochemical reaction of **CLP**, Fe_2O_3 and Oxone®.

Instrument Name LCMS | **Data Filename** D:\Chem32\1\Data\2102\21031605.D
Acq Method Andres - SCAN Pos_m | **Sample Name** PS121 OXONE
DA Method HRMS.m | **Position** Vial 44
User Name SYSTEM | **Comment** ACN+0,1%HCOOH/H2O+0.1%HCOOH Gradient

User Chromatograms

Fragmentor Voltage 0 **Collision Energy** 0 **Ionization Mode** ESI

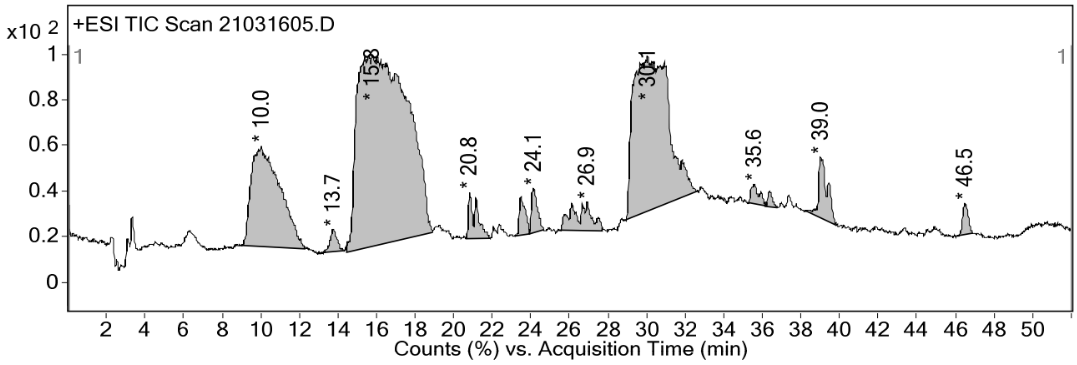

Integration Peak List

Peak	Start	RT	End	Height	Area	Area %
1	8.9	10	12.3	9791679	1008171598	29.84
2	13.1	13.7	14.3	2116221	52831078	1.56
3	14.5	15.8	18.9	18640508	3378058620	100
4	20.6	20.8	21.9	4474662	127933581	3.79
5	23.1	23.5	23.9	3720979	79787525	2.36
6	23.9	24.1	24.7	4300057	90914437	2.69
7	25.5	26.9	27.7	2740069	184110048	5.45
8	28.8	30.1	32.7	15056276	1920662089	56.86
9	38.2	39	39.9	5885295	190812843	5.65
10	46.1	46.5	46.9	2997807	59448003	1.76

Figure S8. LC-MS data of mixture obtained after mechanochemical reaction of **CLP**, TiO_2 and Oxone®.

*Mechanical oxidative degradation of Plavix® (Clopidogrel hydrogen sulfate, **CLP**)*

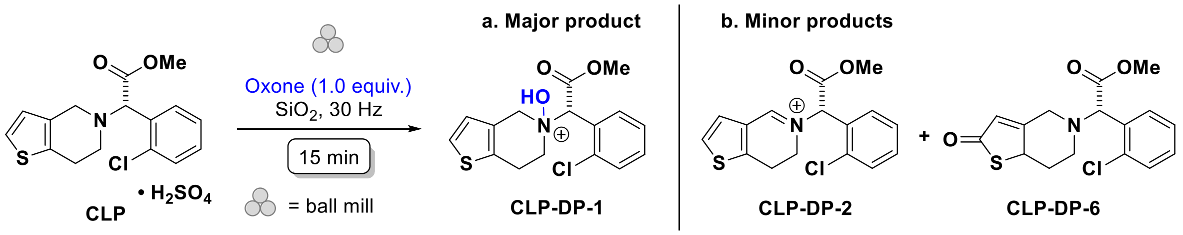

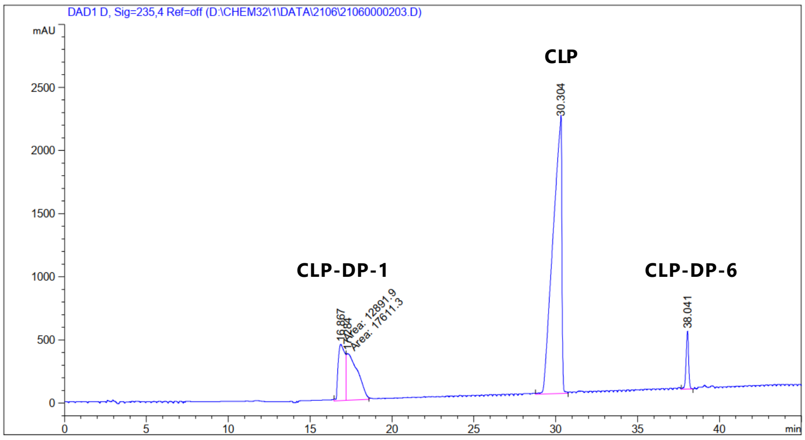

Figure S9. HPLC trace of a sample obtained after mechanochemical reaction of Plavix® with Oxone®.

**Instrument Name**	LCMS	**Data Filename**	D:\Chem32\1\Data\2105\21060304.D
**Acq Method**	Andres - SCAN Pos_m	**Sample Name**	PS 127 OXONE
**DA Method**	HRMS.m	**Position**	Vial 43
**User Name**	SYSTEM	**Comment**	ACN+0,1%HCOOH/H2O+0.1%HCOOH Gradient

User Chromatograms

Fragmentor Voltage 0 **Collision Energy** 0 **Ionization Mode** ESI

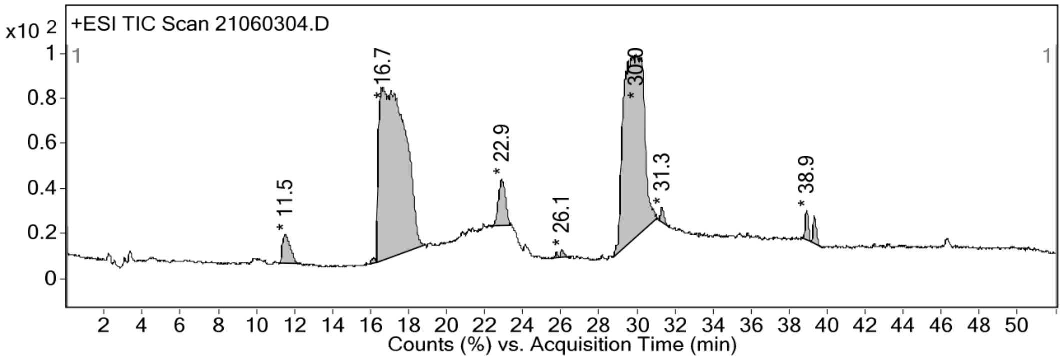

Integration Peak List

Peak	Start	RT	End	Height	Area	Area %
1	11.2	11.5	12.2	2370098	65521562	4.83
2	15.9	16.2	16.3	403729	5800042	0.43
3	16.3	16.7	18.8	14206445	1357508371	100
4	22.3	22.9	23.4	3853703	96738375	7.13
5	25.5	26.1	26.6	623211	8713664	0.64
6	28.7	30	31.1	15080157	1154914073	85.08
7	31.1	31.3	31.7	1197839	15806555	1.16
8	38.6	38.9	39.1	2410802	27084186	2
9	39.1	39.3	39.7	2249135	26856466	1.98

Figure S10. LC-MS data of mixture obtained after mechanochemical reaction of Plavix® with Oxone®.

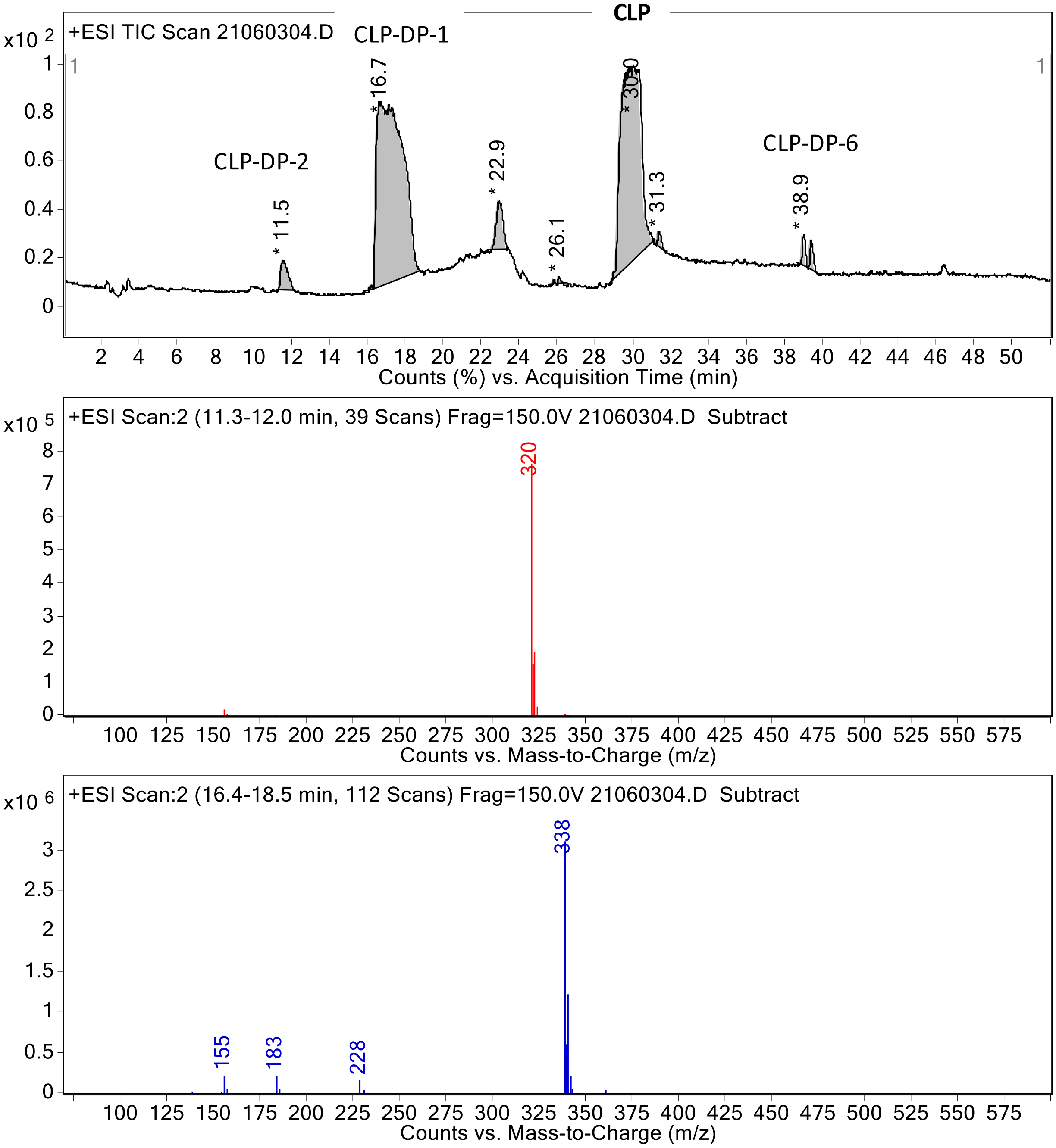

Figure S11. Full LC-MS data of mixture obtained after mechanochemical reaction of Plavix® with Oxone®. Shown are the total ion chromatograms (TIC) along with the DAD response and the MS data at given retention times.

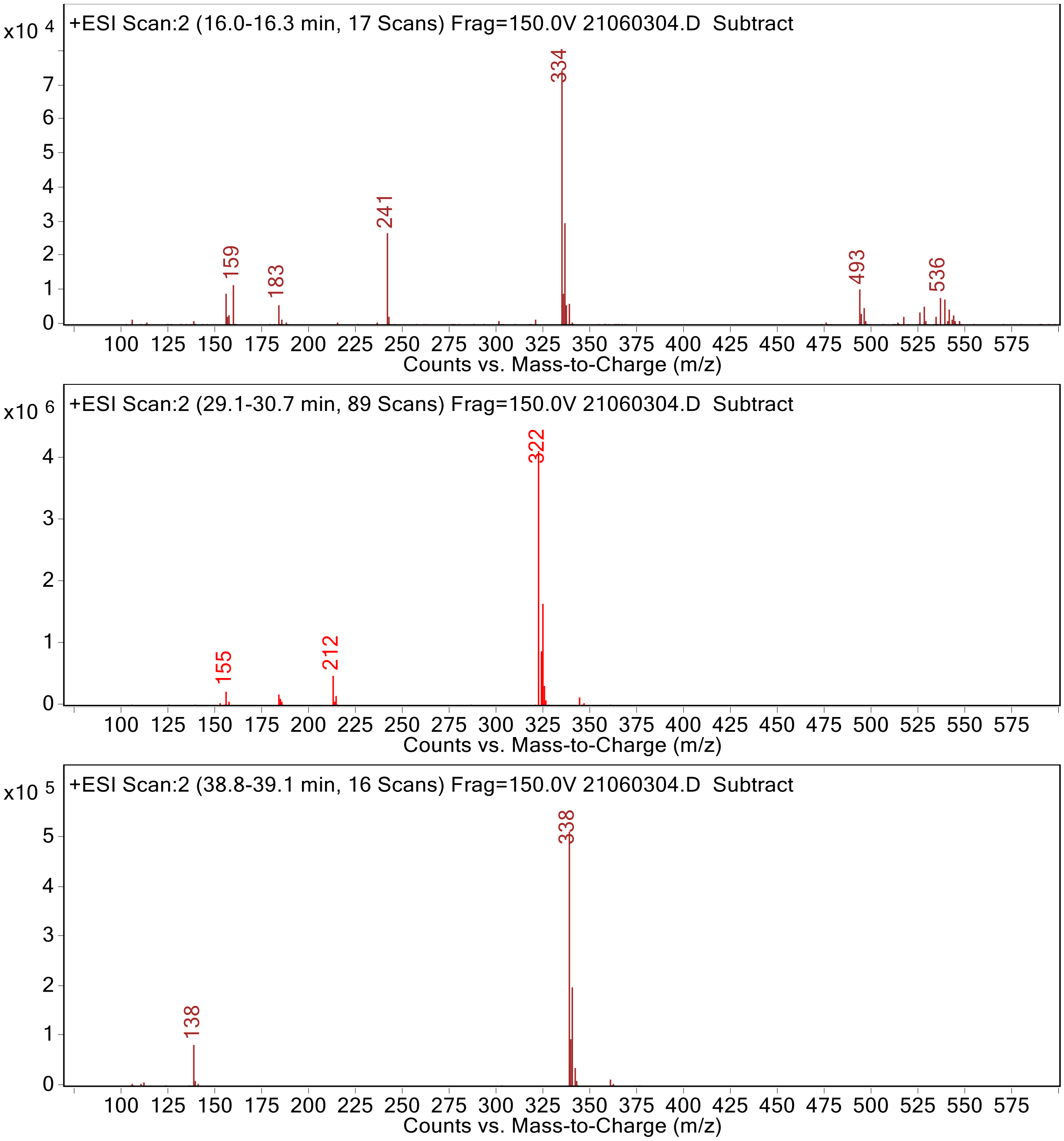

Figure S11, continued. Full LC-MS data of mixture obtained after mechanochemical reaction of Plavix® with Oxone®. Shown are the total ion chromatograms (TIC) along with the DAD response and the MS data at given retention times.

Table S3. The structures of **CLP** (drug product) and its <u>major</u> degradation products.

Entry	Name and Comp. Nr.	Ret. Time	UV spectrum	Mol. Form. Exact Mass	Structure	LCMS (m/z)
**1**	CLP *N*-oxide diastereomers (CLP-DP-1)	16.8	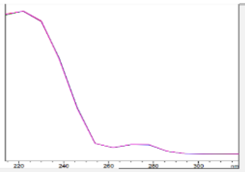	$C_{16}H_{16}ClNO_3S$ 337,0539	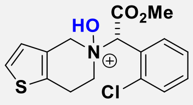	338
**2**	CLP *N*-oxide diastereomers (CLP-DP-1)	17.3	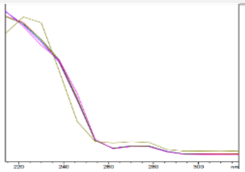	$C_{16}H_{16}ClNO_3S$ 337,0539	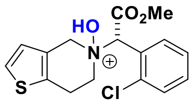	338
**3**	Clopidogrel (**CLP**)	30.3	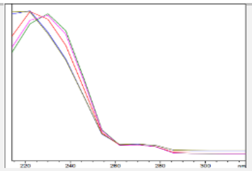	$C_{16}H_{16}ClNO_2S$ 321,0590 (free base)	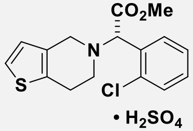	322
**4**	2-oxo-clopidogrel (CLP-DP-6)	38.0	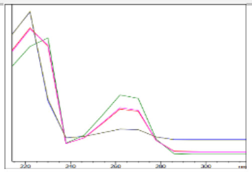	$C_{16}H_{16}ClNO_3S$ 337,0539	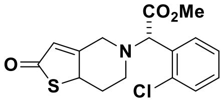	338[1,2,3]

*Mechanical oxidative degradation of Ticlopidin-neuraxpharm® (Ticlopidine hydrochloride, **TIC**)*

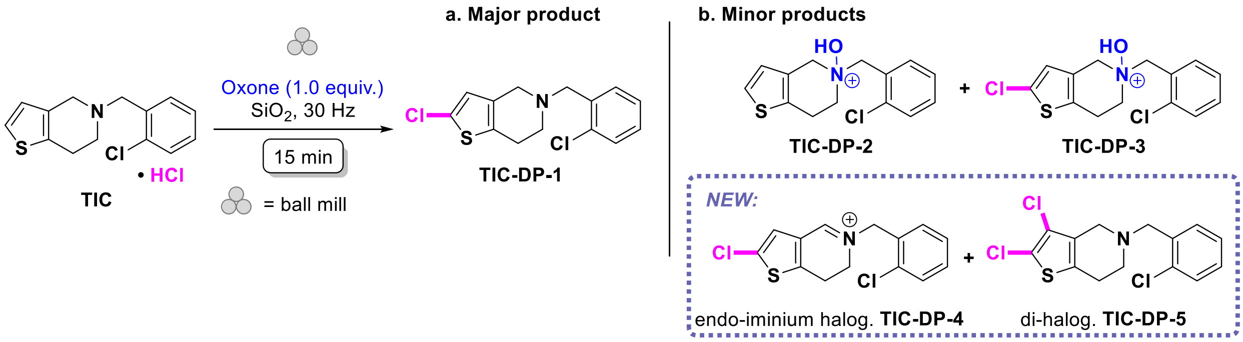

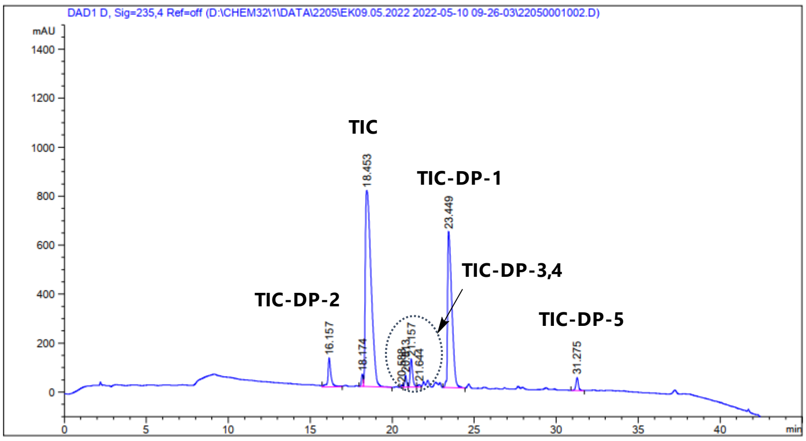

Figure S12. HPLC trace of a sample obtained after mechanochemical reaction of Ticlopidin-neuraxpharm® with Oxone®.

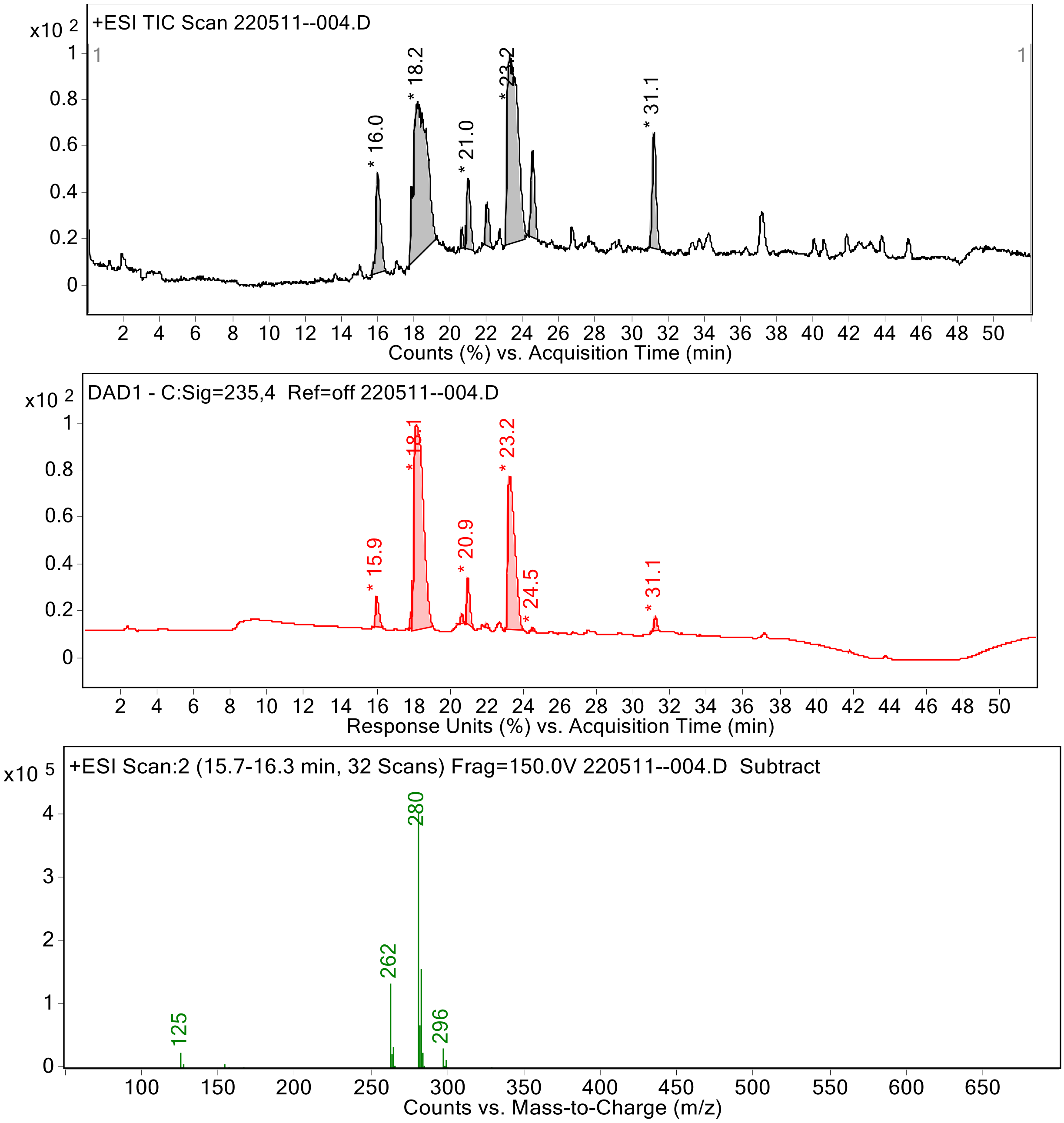

Figure S13. Full LC-MS data of mixture obtained after mechanochemical reaction of Ticlopidin-neuraxpharm® with Oxone®. Shown are the total ion chromatograms (TIC) along with the DAD response and the MS data at given retention times.

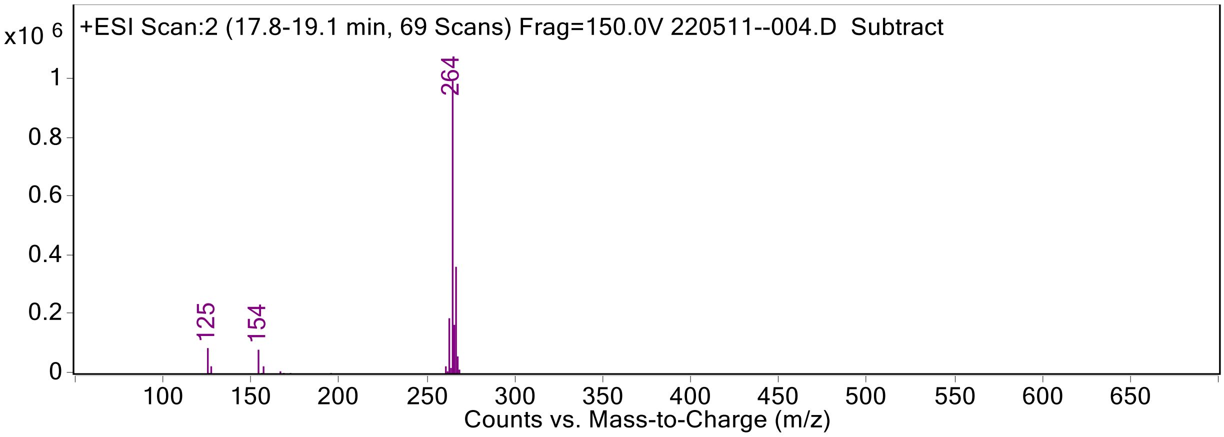

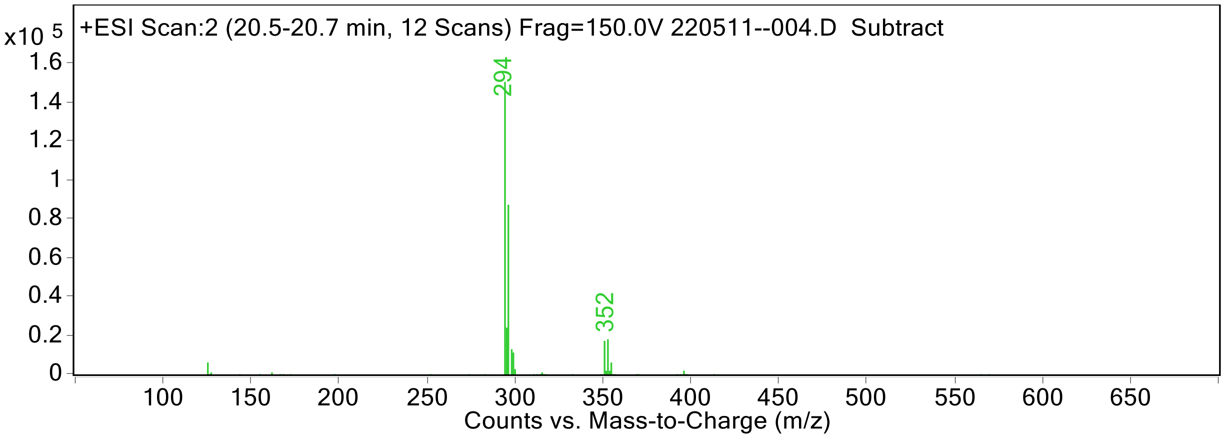

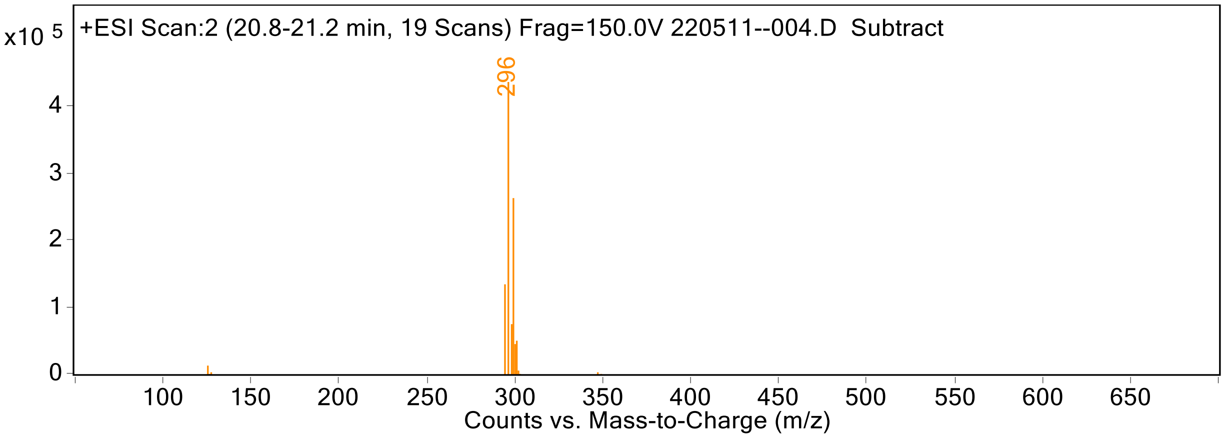

Figure S13, continued. Full LC-MS data of mixture obtained after mechanochemical reaction of Ticlopidin-neuraxpharm® with Oxone®. Shown are the total ion chromatograms (TIC) along with the DAD response and the MS data at given retention times.

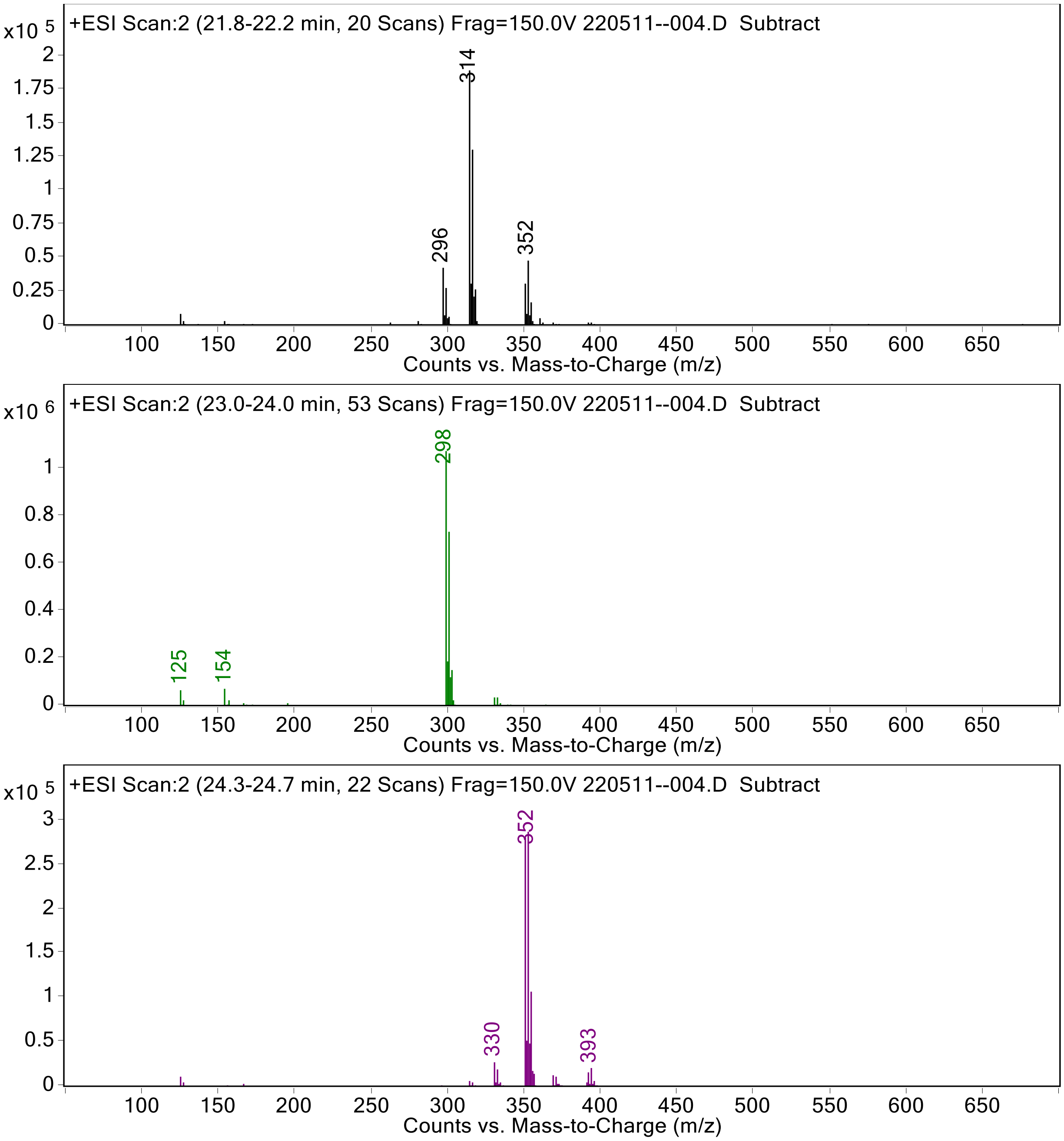

Figure S13, continued. Full LC-MS data of mixture obtained after mechanochemical reaction of Ticlopidin-neuraxpharm® with Oxone®. Shown are the total ion chromatograms (TIC) along with the DAD response and the MS data at given retention times.

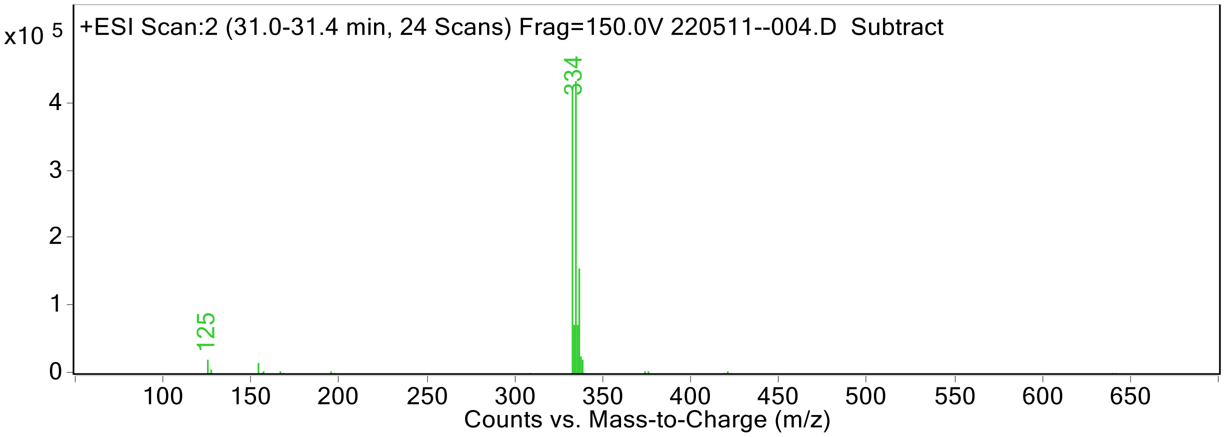

Figure S13, continued. Full LC-MS data of mixture obtained after mechanochemical reaction of Ticlopidin-neuraxpharm® with Oxone®. Shown are the total ion chromatograms (TIC) along with the DAD response and the MS data at given retention times.

Table S4. The structures of **TIC** (drug product) and its degradation products.

Entry	Name and Comp. Nr.	Ret. Time	UV spectrum	Mol. Form. Exact Mass	Structure	LCMS (m/z)
**1**	TIC *N*-oxide (TIC-DP-2)	15.9	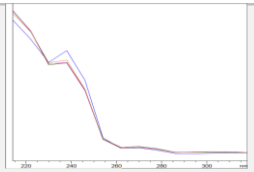	$C_{14}H_{15}ClNOS^{+}$ 280.0557	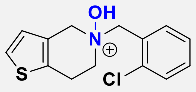	280
**2**	Ticlopidine (**TIC**)	18.1	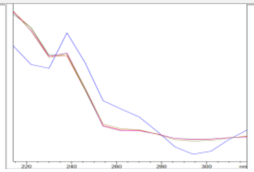	$C_{14}H_{14}ClNS$ 263.0535	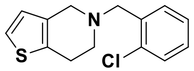	264
**3**	TIC endo-iminium (TIC-DP-4)	20.9	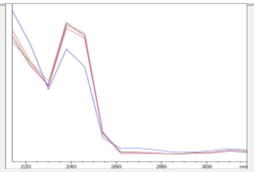	$C_{14}H_{12}Cl_2NS^{+}$ 296.0062	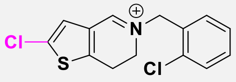	296
**4**	TIC chlor. *N*-oxide (TIC-DP-3)	23.2	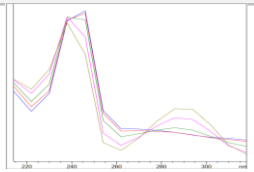	$C_{14}H_{14}Cl_2NOS^{+}$ 314.0168	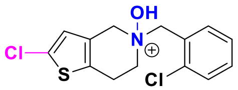	314
**5**	TIC chlor. (TIC-DP-1)	24.5	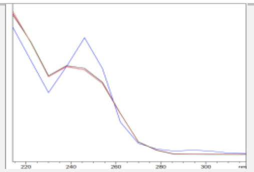	$C_{14}H_{13}Cl_2NS$ 297.0146	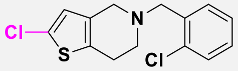	298
**6**	TIC di-chlor. (TIC-DP-5)	31.1	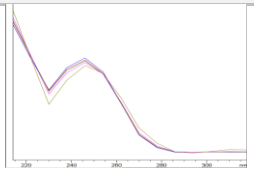	$C_{14}H_{12}Cl_3NS$ 330.9756	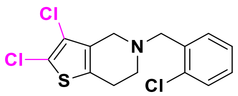	330

*Mechanical oxidative degradation of Efient® (Prasugrel hydrochloride, **PRA**)*

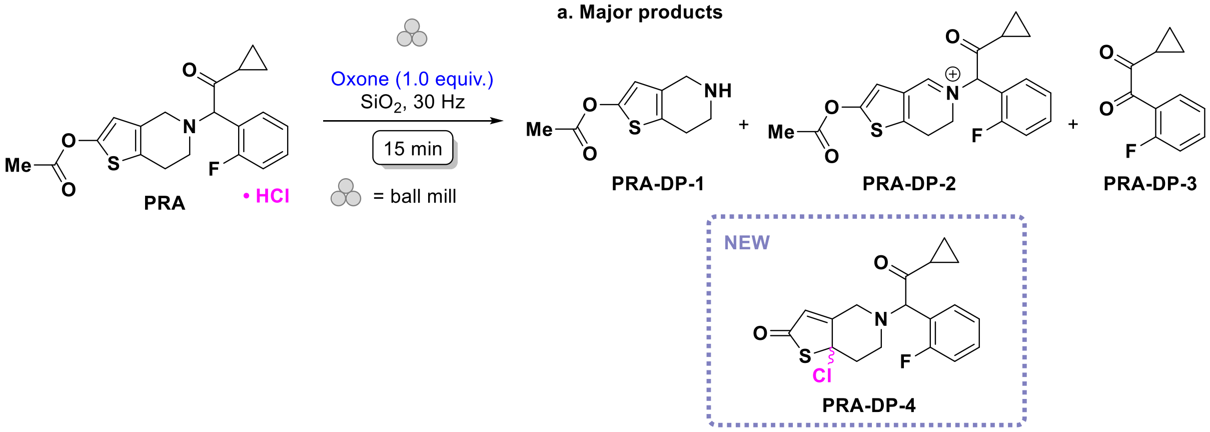

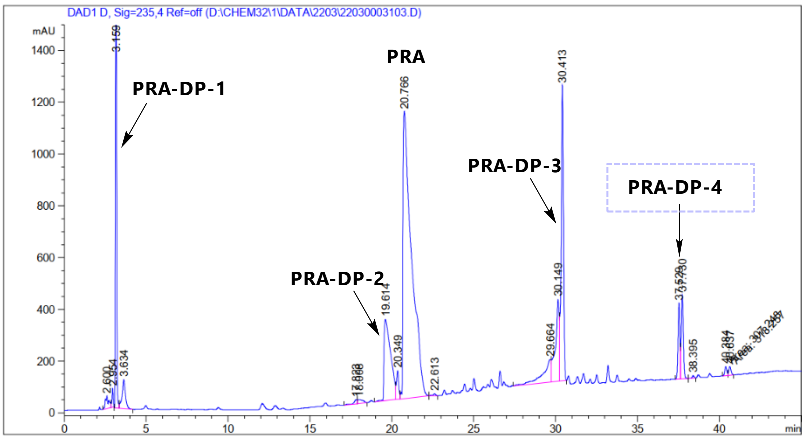

Figure S14. HPLC trace of a sample obtained after mechanochemical reaction of Efient® with Oxone®.

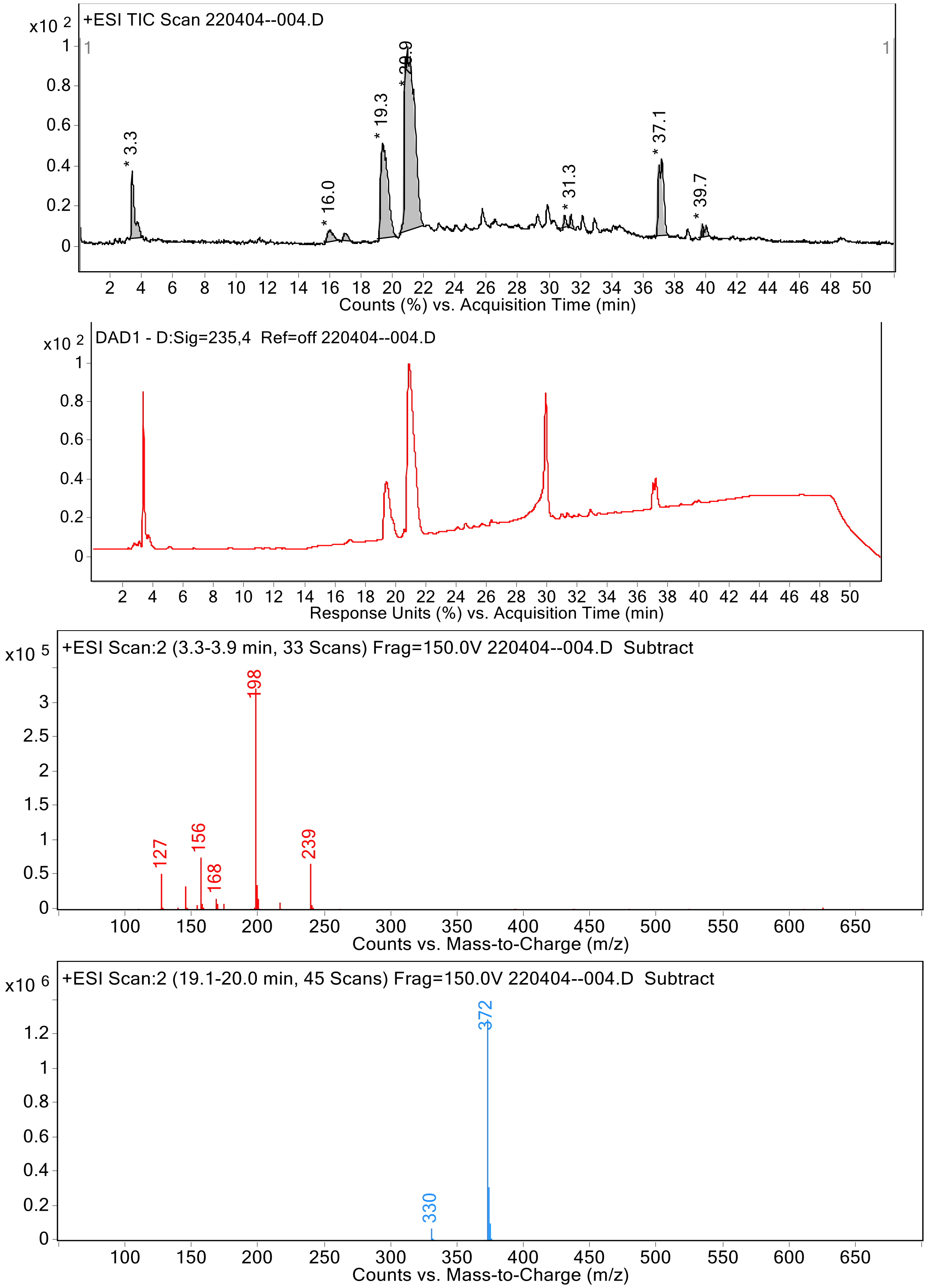

Figure S15. Full LC-MS data of mixture obtained after mechanochemical reaction of Efient® with Oxone®. Shown are the total ion chromatograms (TIC) along with the DAD response and the MS data at given retention times.

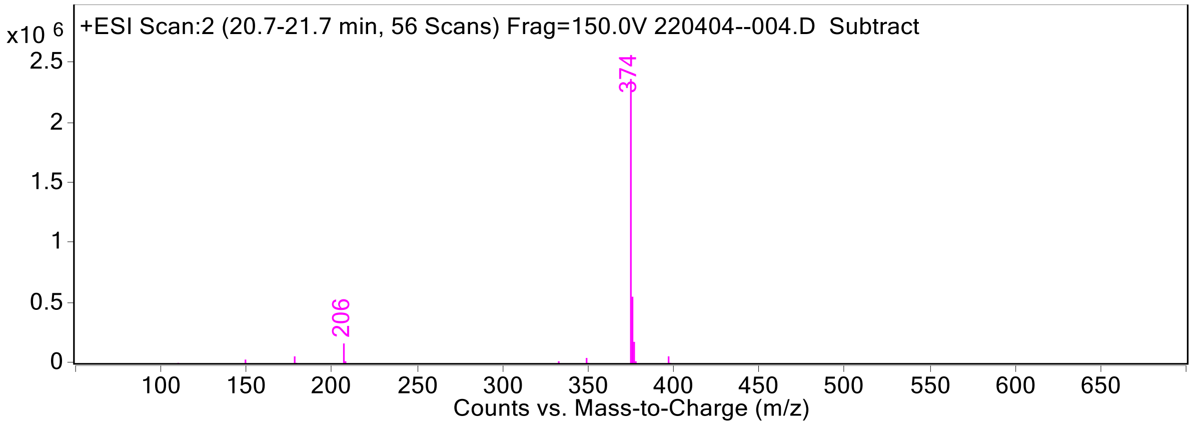

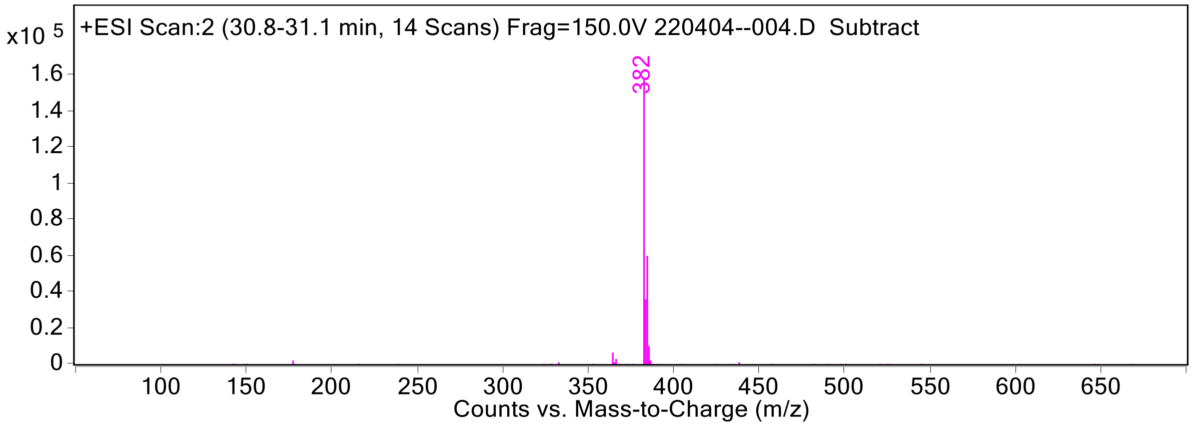

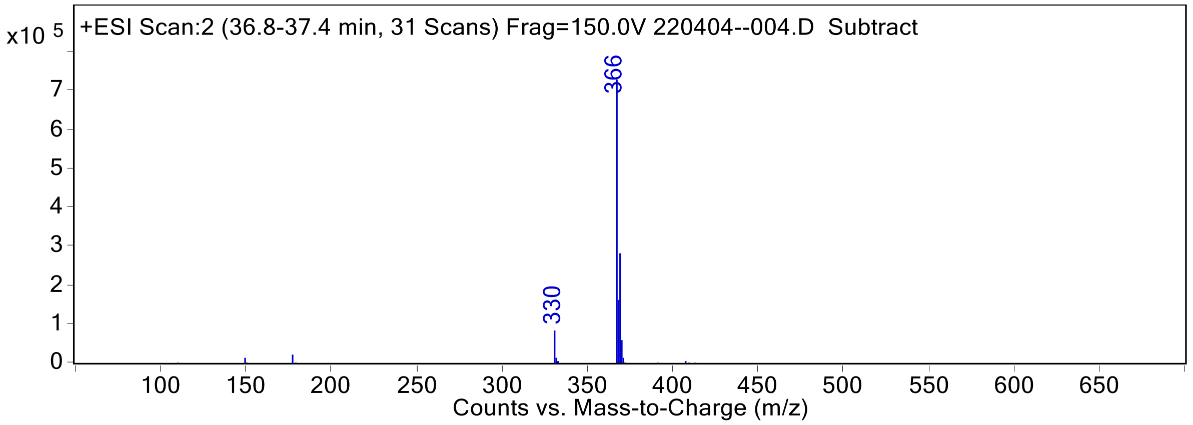

Figure S15, continued. Full LC-MS data of mixture obtained after mechanochemical reaction of Efient® with Oxone®. Shown are the total ion chromatograms (TIC) along with the DAD response and the MS data at given retention times.

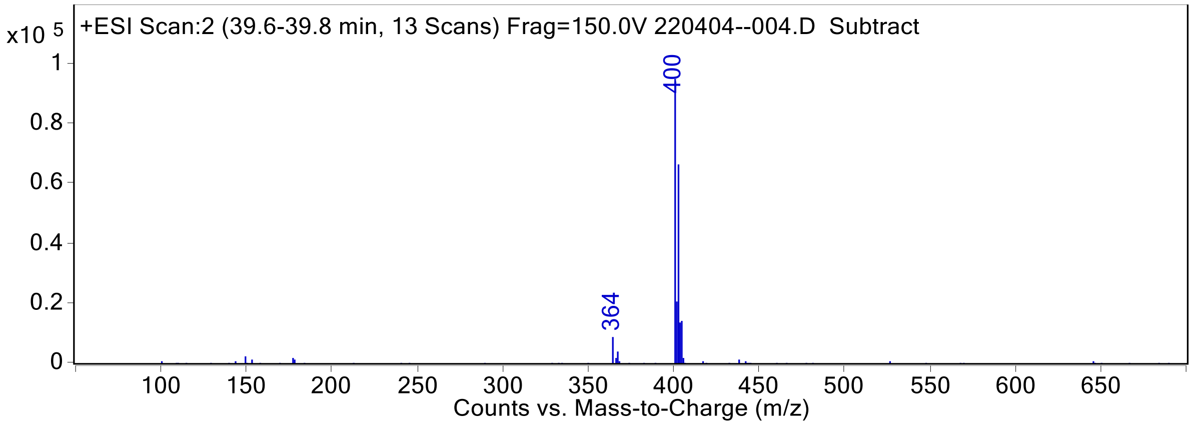

Figure S15, continued. Full LC-MS data of mixture obtained after mechanochemical reaction of Efient® with Oxone®. Shown are the total ion chromatograms (TIC) along with the DAD response and the MS data at given retention times.

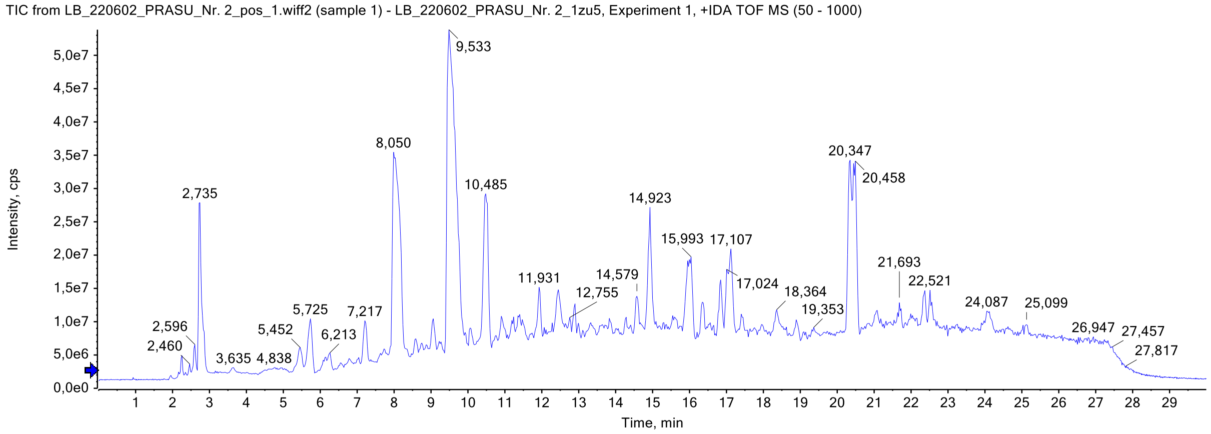

Figure S16. LC-ESI-HRMS of of mixture obtained after mechanochemical reaction of Efient® with Oxone®.

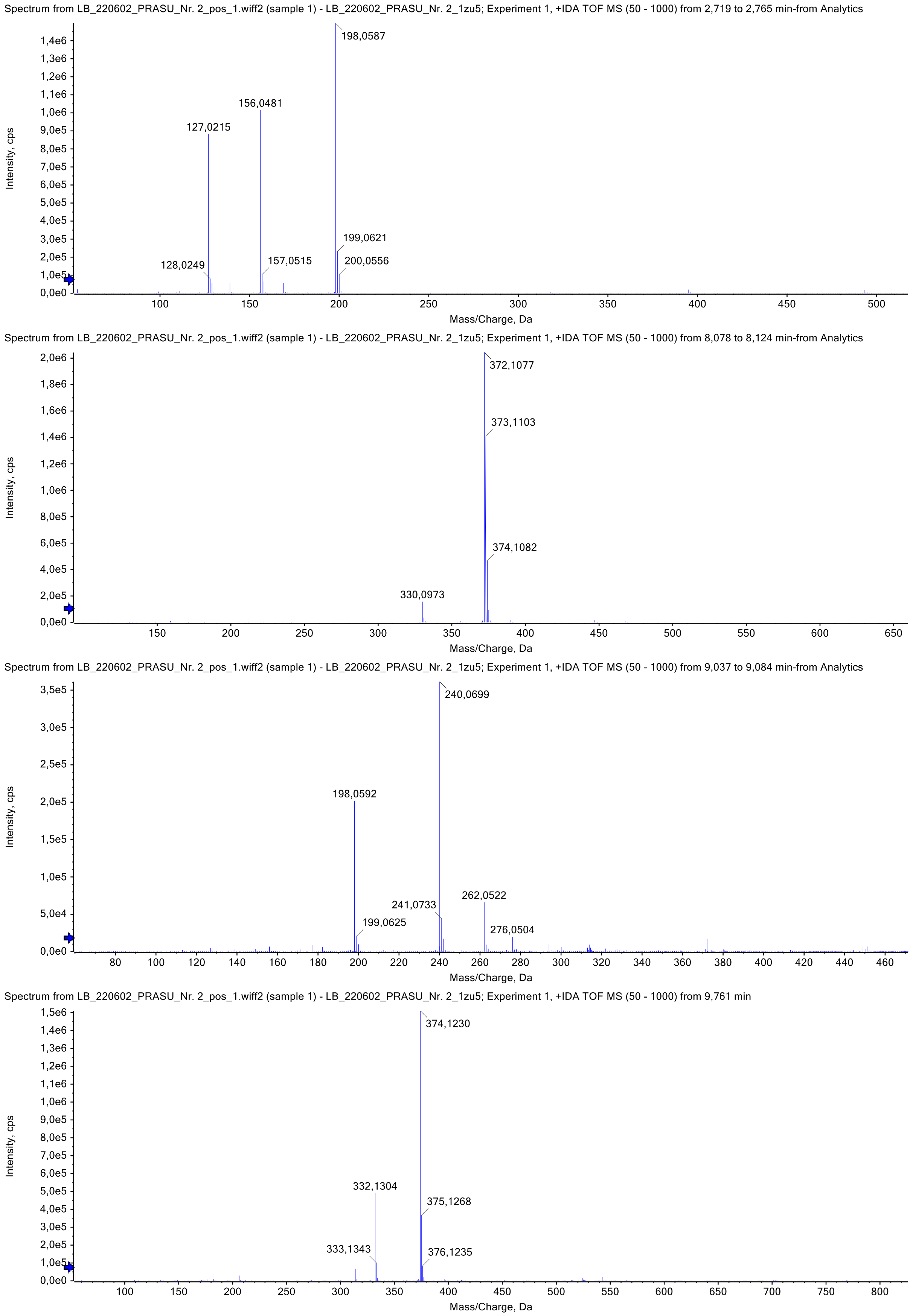

Figure S16, continued. LC-ESI-HRMS of of mixture obtained after mechanochemical reaction of Efient® with Oxone®.

Spectrum from LB_220602_PRASU_Nr. 2_pos_1.wiff2 (sample 1) - LB_220602_PRASU_Nr. 2_1zu5; Experiment 1, +IDA TOF MS (50 - 1000) from 10,388 min

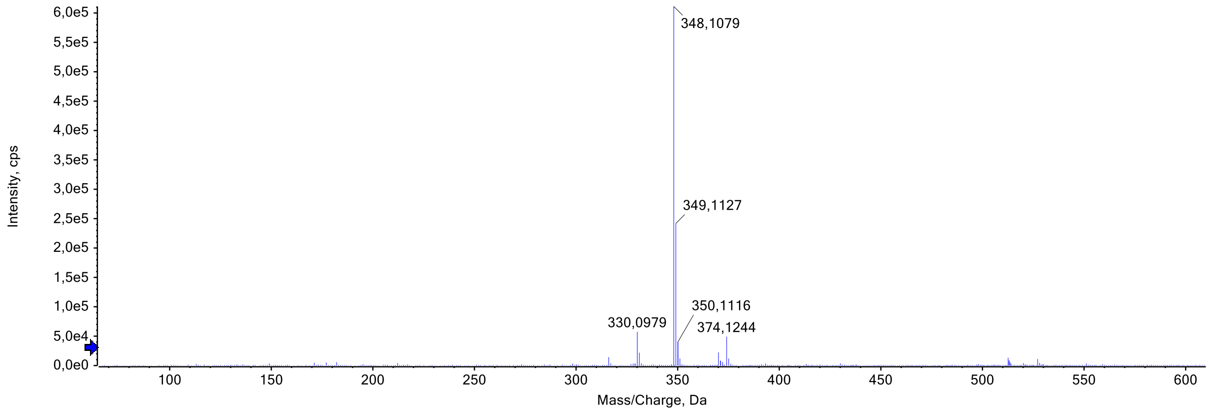

Spectrum from LB_220602_PRASU_Nr. 2_pos_1.wiff2 (sample 1) - LB_220602_PRASU_Nr. 2_1zu5; Experiment 1, +IDA TOF MS (50 - 1000) from 13,877 to 13,925 min-from Analytics

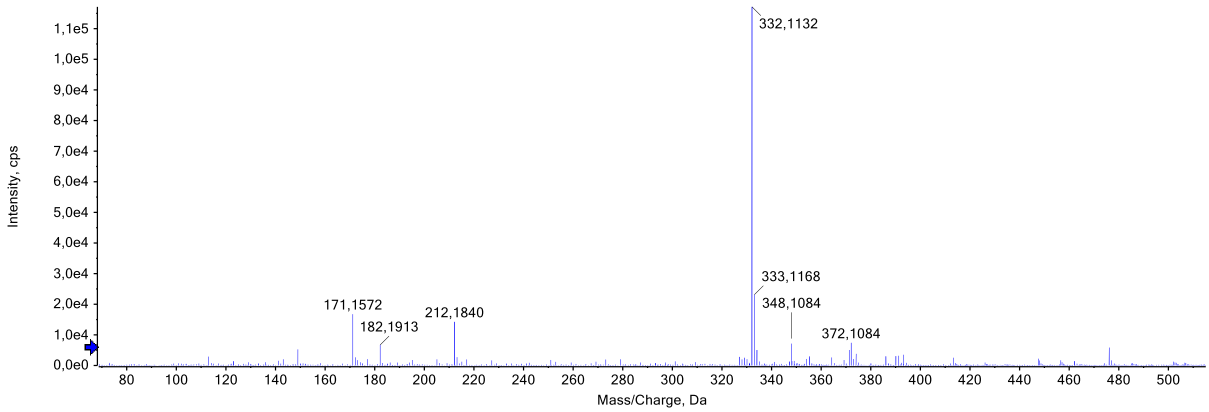

Spectrum from LB_220602_PRASU_Nr. 2_pos_1.wiff2 (sample 1) - LB_220602_PRASU_Nr. 2_1zu5; Experiment 1, +IDA TOF MS (50 - 1000) from 14,906 to 14,953 min-from Analytics

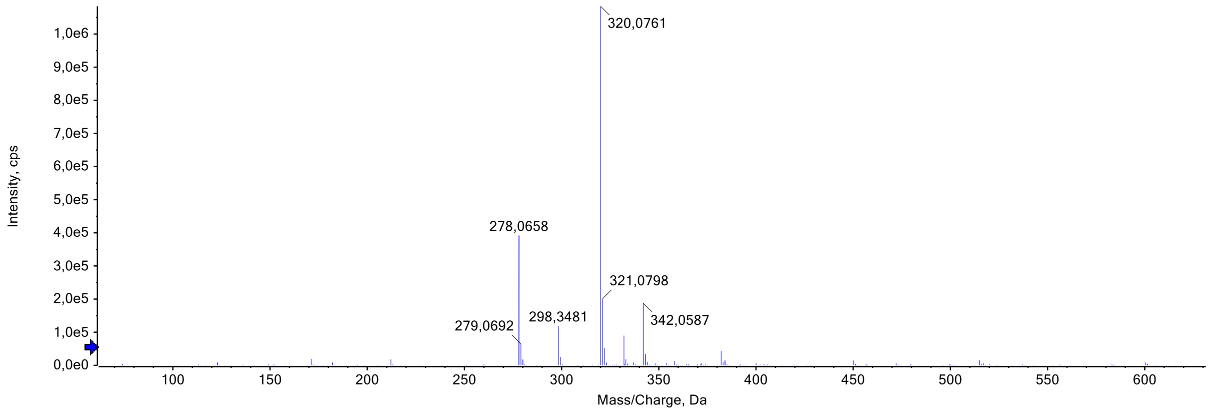

Spectrum from LB_220602_PRASU_Nr. 2_pos_1.wiff2 (sample 1) - LB_220602_PRASU_Nr. 2_1zu5; Experiment 1, +IDA TOF MS (50 - 1000) from 16,820 to 16,866 min-from Analytics

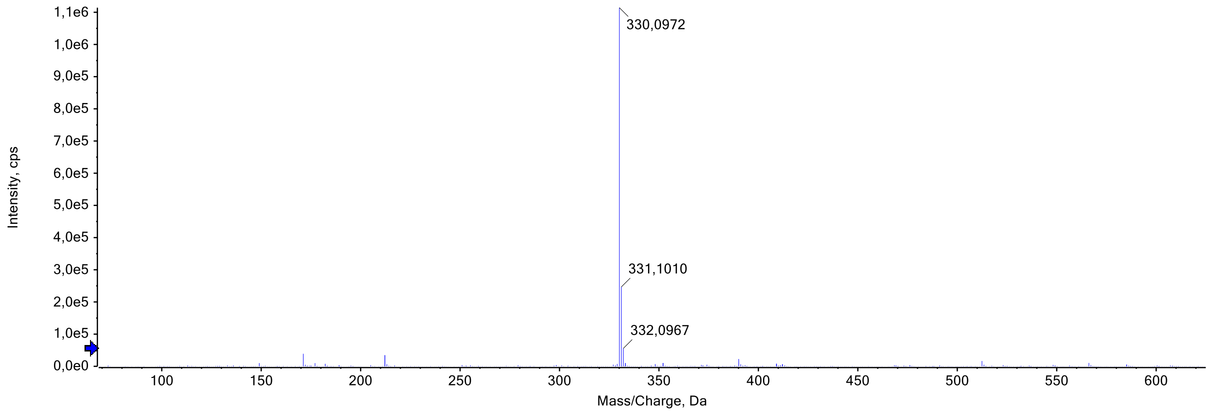

Figure S16, continued. LC-ESI-HRMS of of mixture obtained after mechanochemical reaction of Efient® with Oxone®.

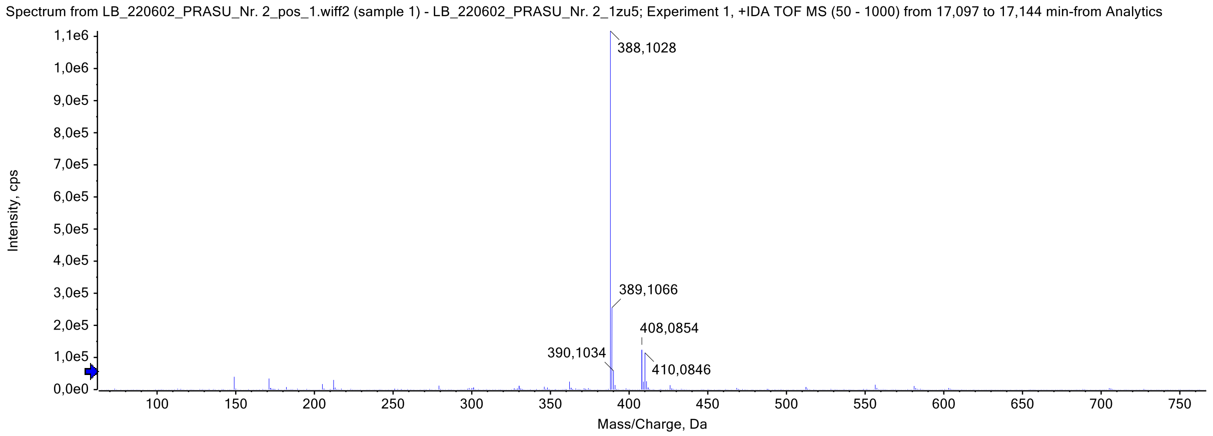

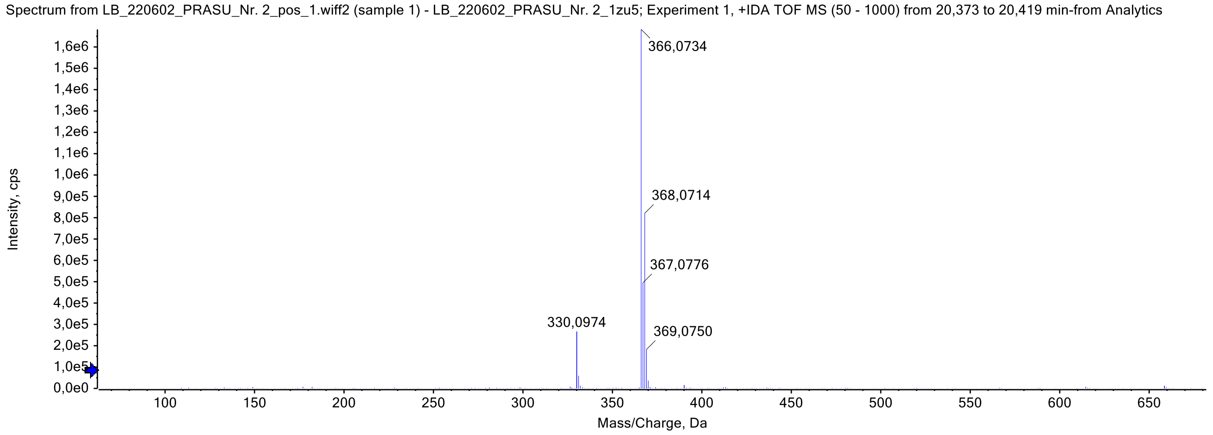

Figure S16, continued. LC-ESI-HRMS of of mixture obtained after mechanochemical reaction of Efient® with Oxone®.

Table S5. HRMS assigned structures of **PRA** (drug product) and its <u>major</u> degradation products.

Entry	Name and Compound Number	Ret. Time HPLC/ LC-ESI-HRMS	UV spectrum	Chemical Formula and Exact Mass	Structure	HRMS (m/z)	Mass error [ppm]
**1**	Thienopyridine acetylated (PRA-DP-1)	3.16 (HPLC) 2.7	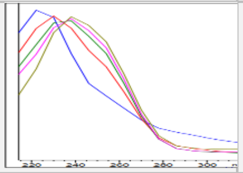	$C_9H_{11}NO_2S$ 197.0510	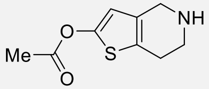	198.0587	1.0
**2**	Endo-iminium (PRA-DP-2)	19.85 (HPLC) 8.2	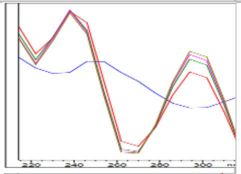	$C_{20}H_{19}FNO_3S^+$ 372.1064	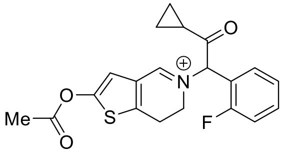	372.1077	1.9
**3**	Prasugrel (**PRA**)	20.76 (HPLC)	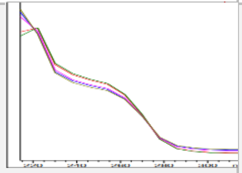	$C_{20}H_{20}FNO_3S$ 373.1148	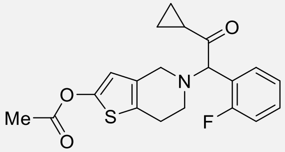	374.1230	1.1
**4**	Diketone (PRA-DP-3)	30.41 (HPLC)	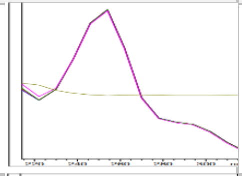	$C_{11}H_9FO_2$ 192.0587	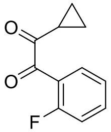	192	-
**5**	PRA-DP-4	37.52 (HPLC) 20.4	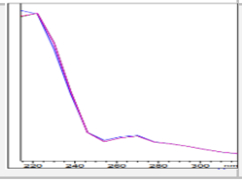	$C_{18}H_{17}ClFNO_2S$ 365.0653	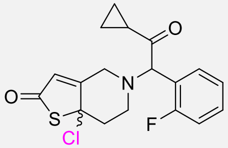	366.0734	0.8

Table S6. HRMS assigned structures of **PRA** and its <u>minor</u> degradation products.

Entry	Name	Ret. Time LC-ESI-HRMS	Molecular Formula and Exact Mass	Structure	HRMS (m/z)	Mass error [ppm]
**1**	PRA-DP-5	9.1	$[C_{11}H_{13}NO_3S+H]^+$ 240.0694	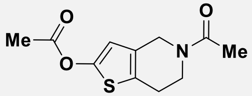	240.0699	2.1
**2**	PRA-DP-6	10.5	$[C_{18}H_{18}FNO_3S+H]^+$ 348.1070	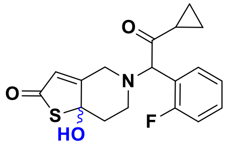	348.1079	2.6
**3**	PRA-DP-7	13.8	$[C_{18}H_{18}FNO_2S+H]^+$ 332.1115	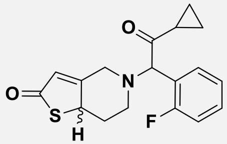	332.1132	3.3
**4**	Fluoro-benzamide (PRA-DP-8)	14.9	$[C_{16}H_{14}FNO_3S+H]^+$ 320.0757	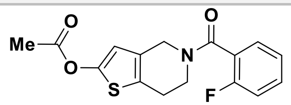	320.0761	1.2
**5**	PRA-DP-9	16.8	$[C_{18}H_{17}FNO_2S]^+$ 330.0964	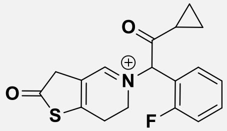	330.0972	2.4
**6**	PRA-DP-10	17.1	$[C_{20}H_{18}FNO_4S+H]^+$ 388.1019	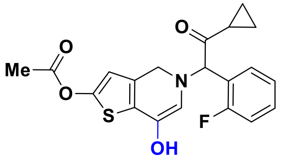	388.1028	2.3

Data File D:\CHEM32\1\DATA\2206\22060002200.D
Sample Name: Mech.Ox.PRA Oxone Fr.40

===

```
Acq. Operator   : SYSTEM                          Seq. Line :   1
Acq. Instrument : LC4                              Location : Vial 1
Injection Date  : 6/22/2022 12:01:19 PM                 Inj :   1
                                                 Inj Volume : 15.000 µl
Different Inj Volume from Sample Entry!  Actual Inj Volume : 5.000 µl
Acq. Method     : C:\CHEM32\1\METHODS\METHOD 2 (UNI WÜ) 24.09.2020.M
Last changed    : 4/20/2022 10:59:54 AM by SYSTEM
Analysis Method : C:\CHEM32\1\METHODS\METHOD 2 (UNI WÜ) 24.09.2020.M
Last changed    : 6/23/2022 11:41:55 AM by SYSTEM
                  (modified after loading)
Method Info     : Zorbax Eclipse Plus -C8    B: ACN+0,1% FA     A: 0.1%FA, Gradient  1ml/min
                  25°C

Additional Info : Peak(s) manually integrated
```

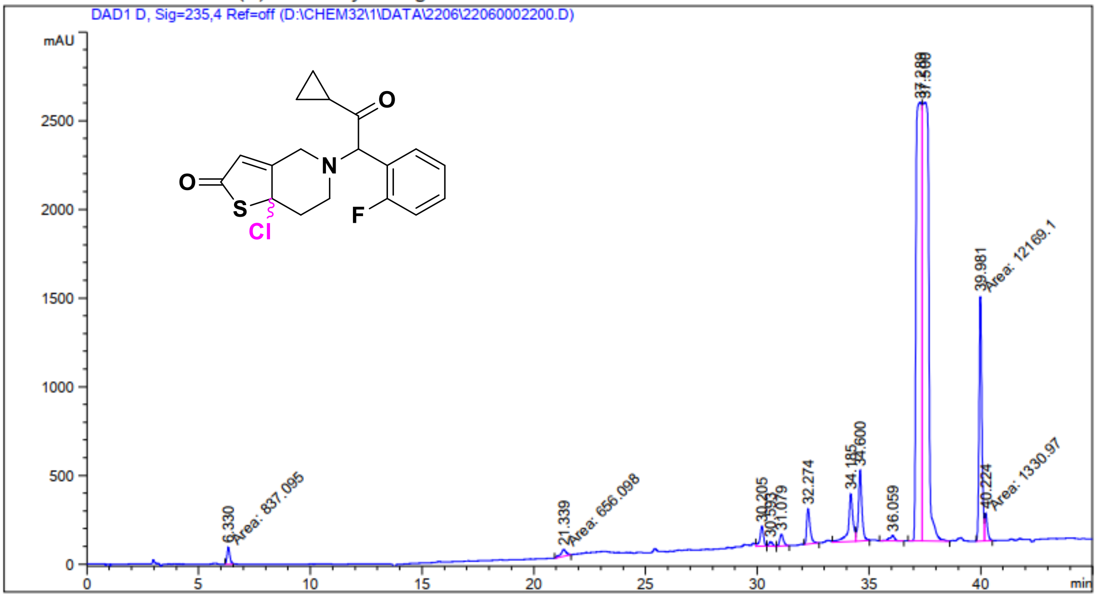

===

Internal Standard Report

===

```
Sorted By          :      Signal
Multiplier         :      1.0000
Dilution           :      1.0000
Do not use Multiplier & Dilution Factor with ISTDs
```

===

Figure S17. Copy of HPLC chromatogram of enriched halogenated product **PRA-DP-4** (77% purity).

NMR data

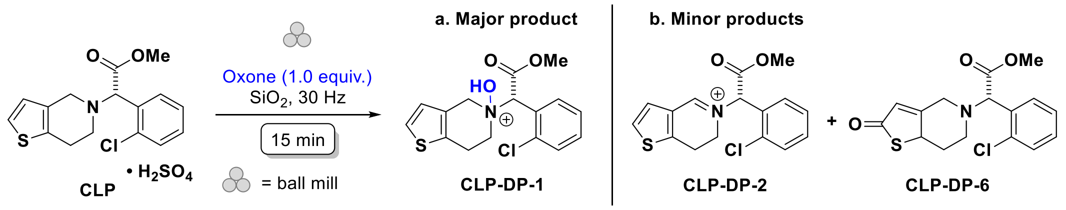

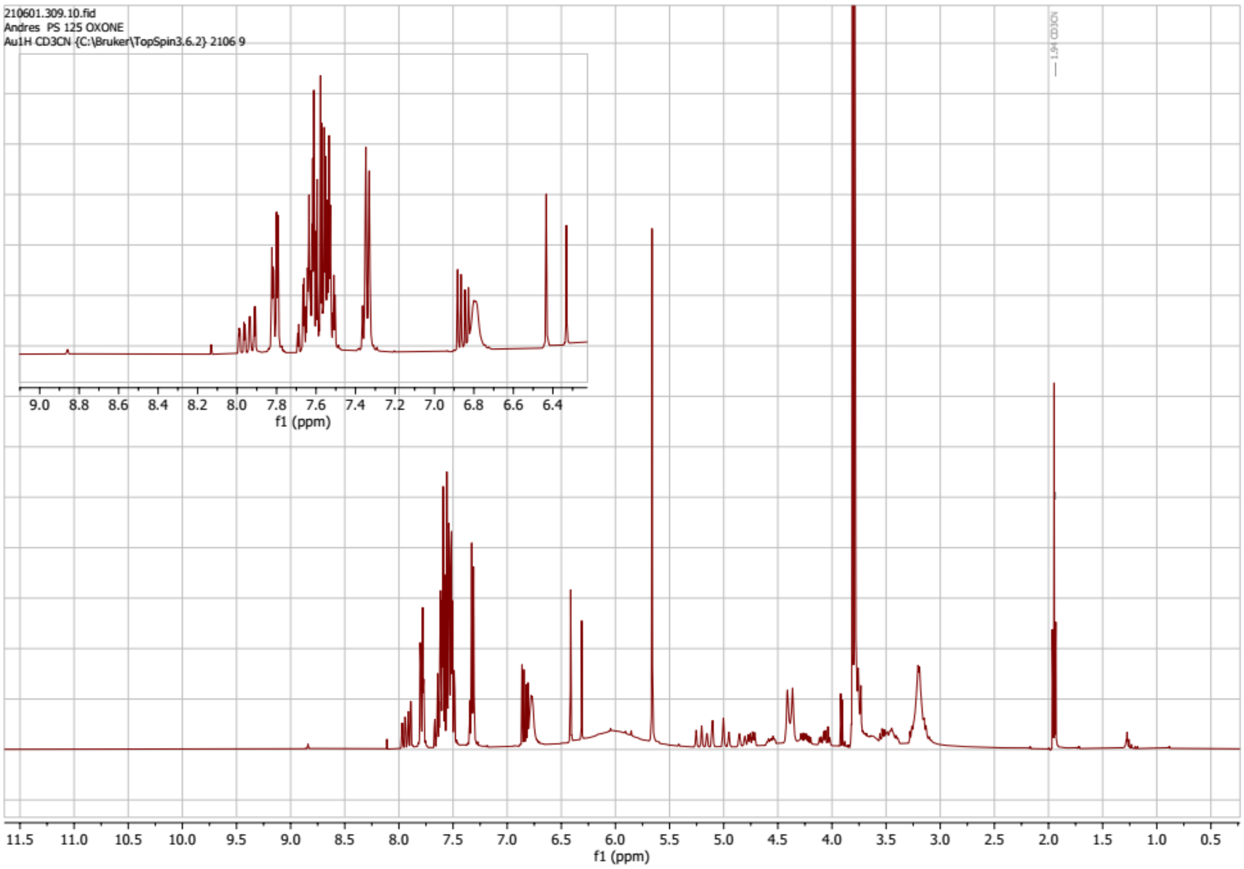

Figure S18. ^{1}H NMR spectrum of crude reaction mixture – Plavix®/Oxone® (CD_3CN, 300 MHz). The inset shows the extension of the aromatic area with characteristic resonances of **CLP-DP-1** and **CLP-DP-2**.

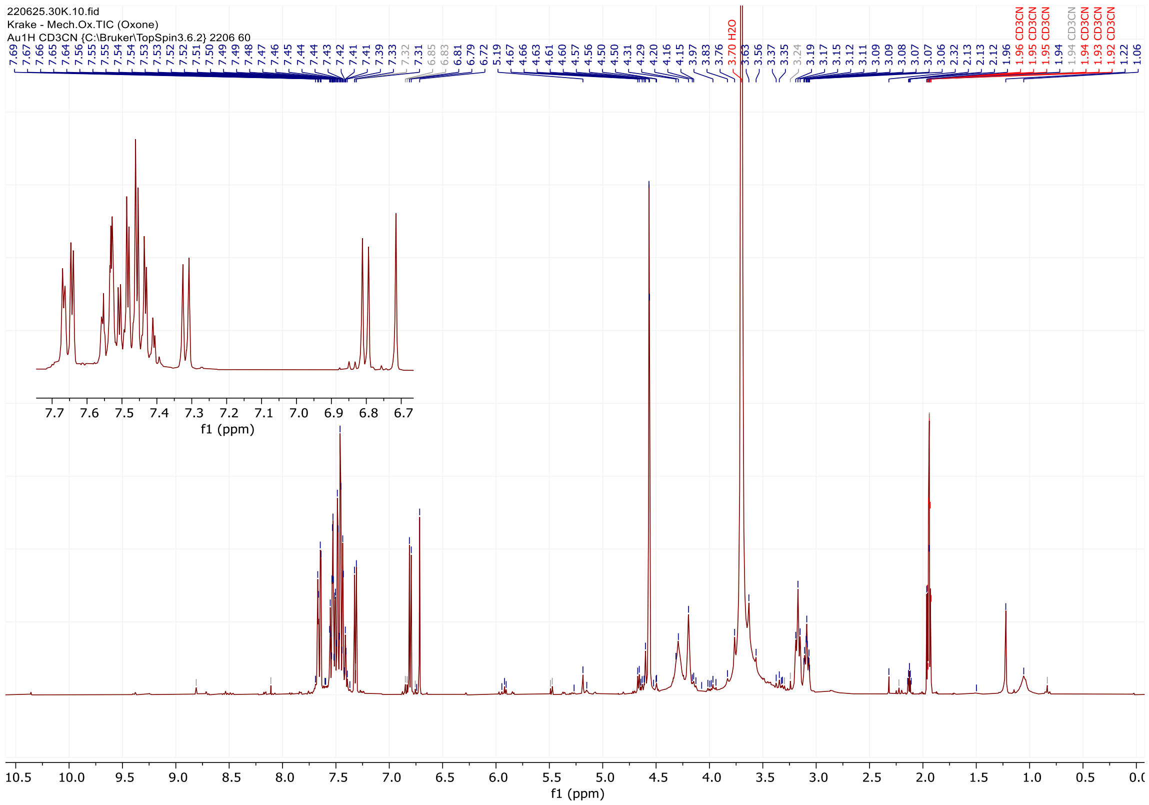

Figure S19. ^{1}H NMR spectrum of crude reaction mixture – Ticlopidin-neuraxpharm/Oxone® (CD_3CN, 300 MHz).

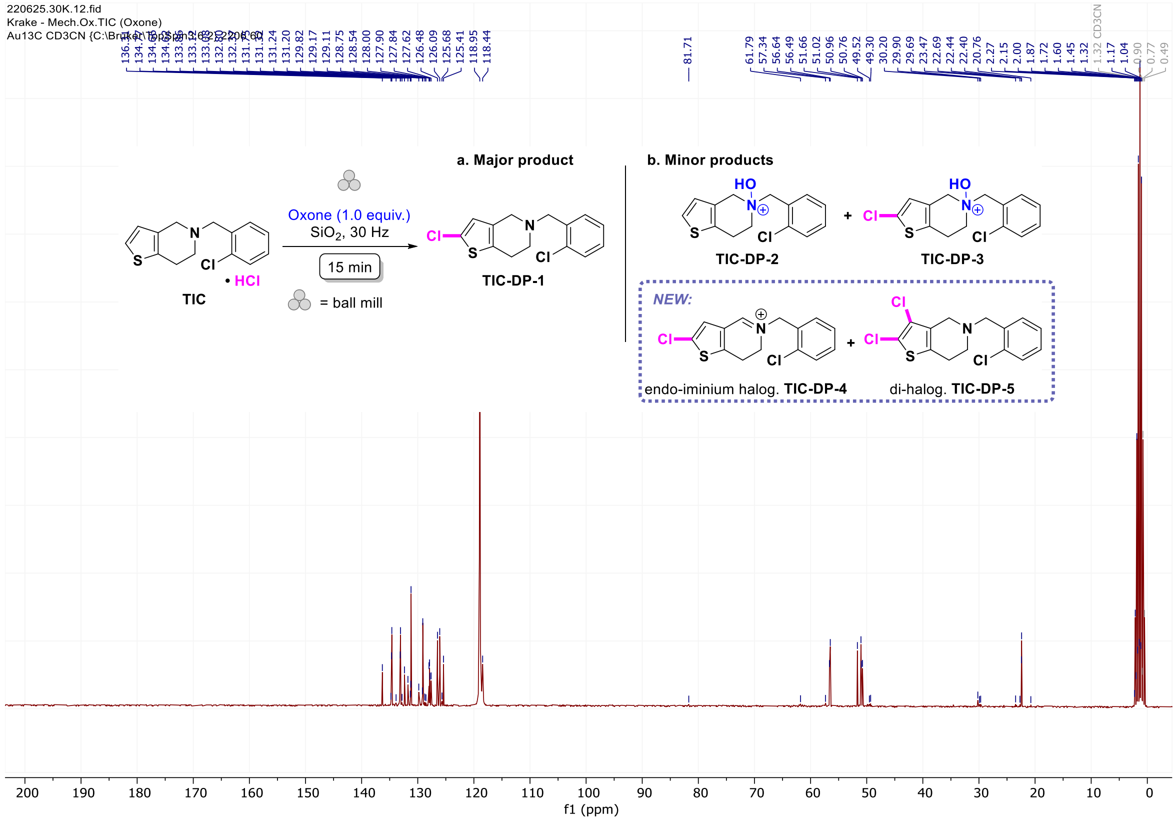

Figure S20. ^{13}C NMR spectrum of crude reaction mixture – Ticlopidin-neuraxpharm/Oxone® (CD_3CN, 75 MHz).

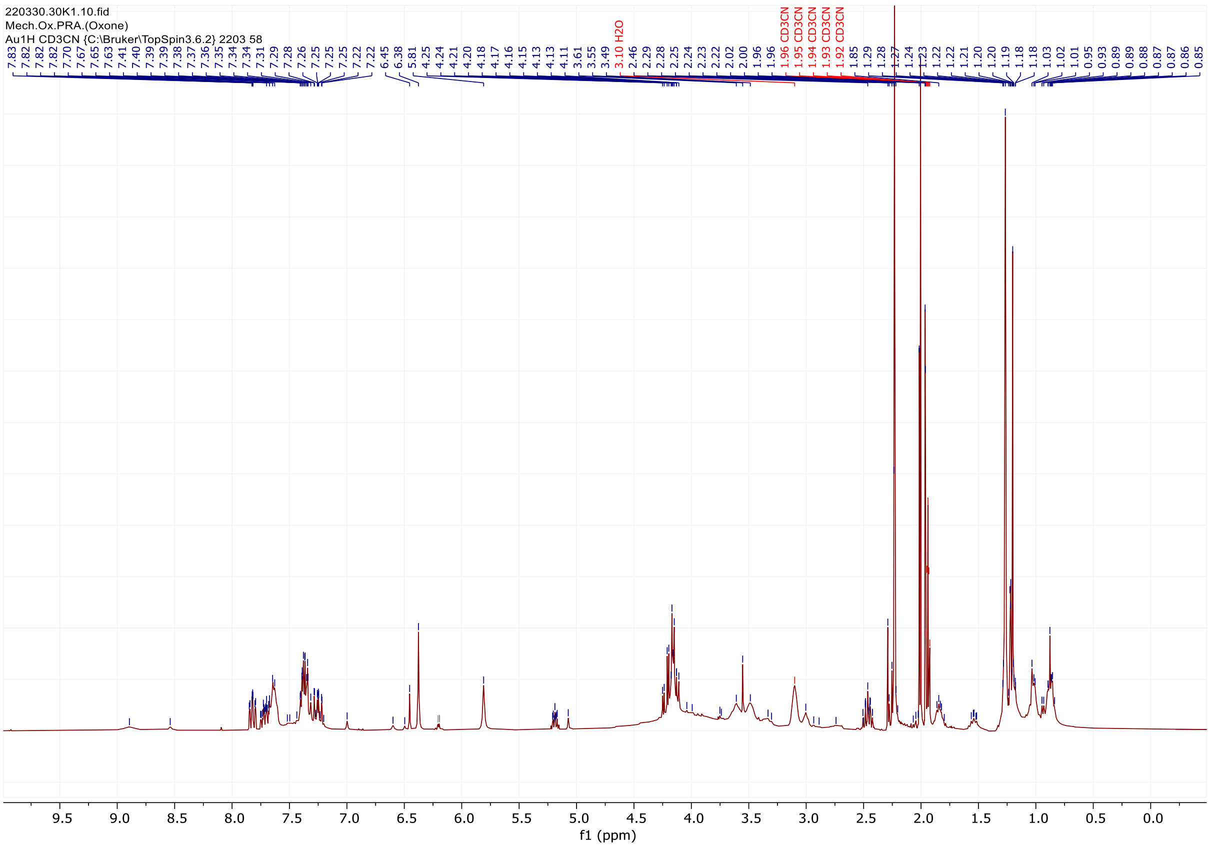

Figure S21. ^{1}H NMR spectrum of crude reaction mixture – Efient®/Oxone® (CD_3CN, 300 MHz).

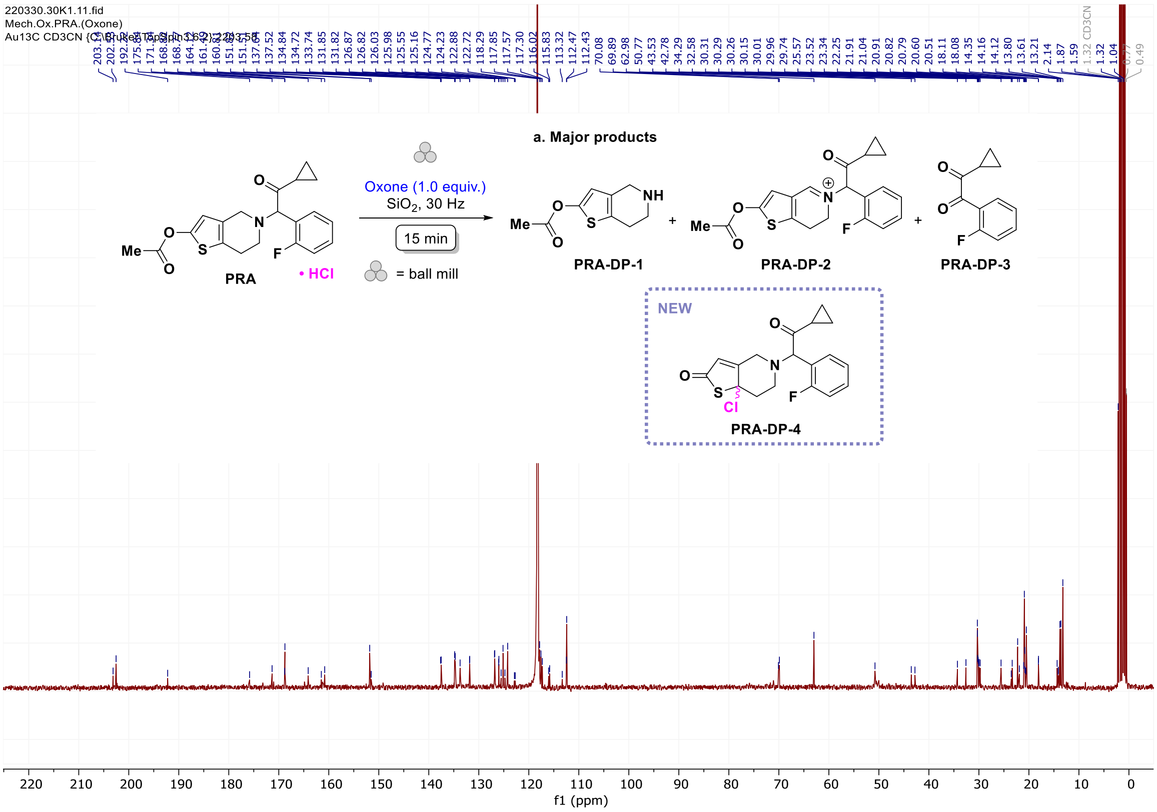

Figure S22. ^{13}C NMR spectrum of crude reaction mixture – Efient®/Oxone® (CD_3CN, 75 MHz).

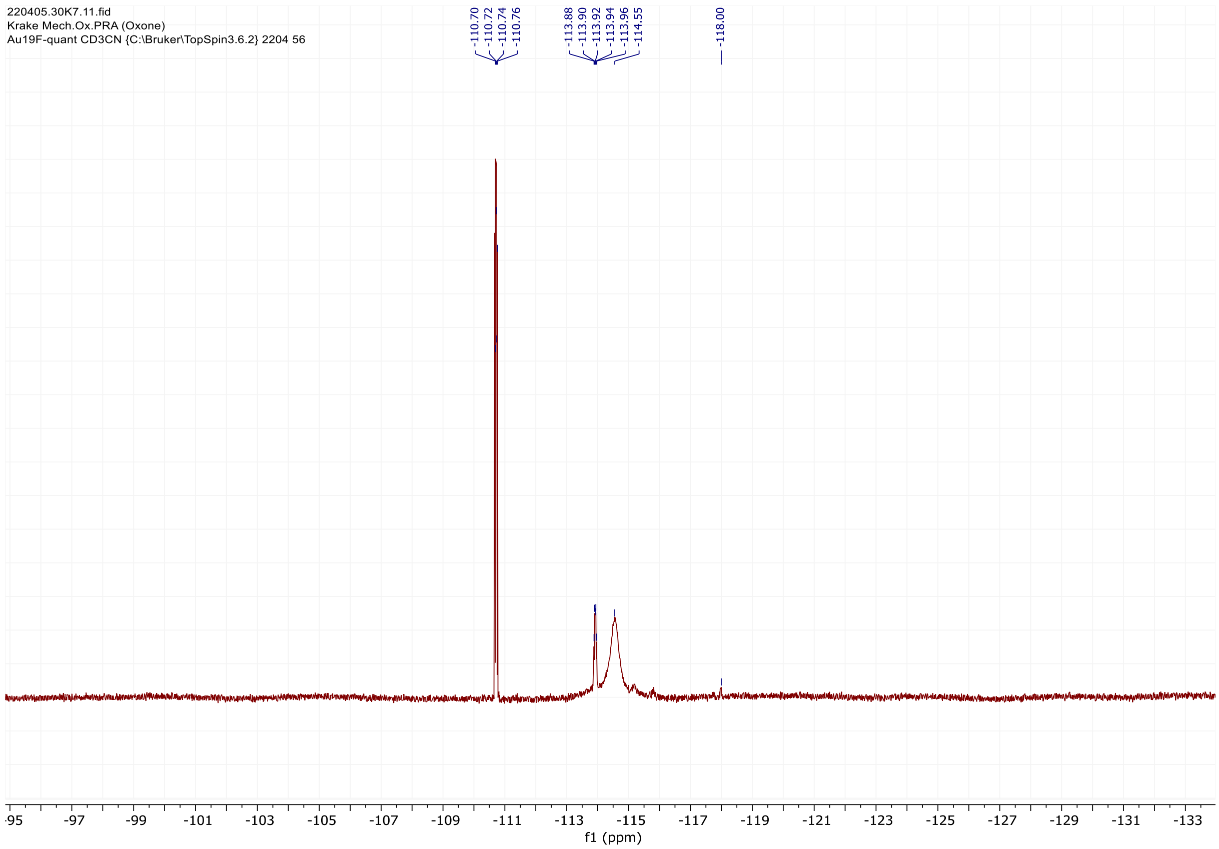

Figure S23. ^{19}F NMR spectrum of crude reaction mixture – Efient®/Oxone® (CD_3CN, 282 MHz).

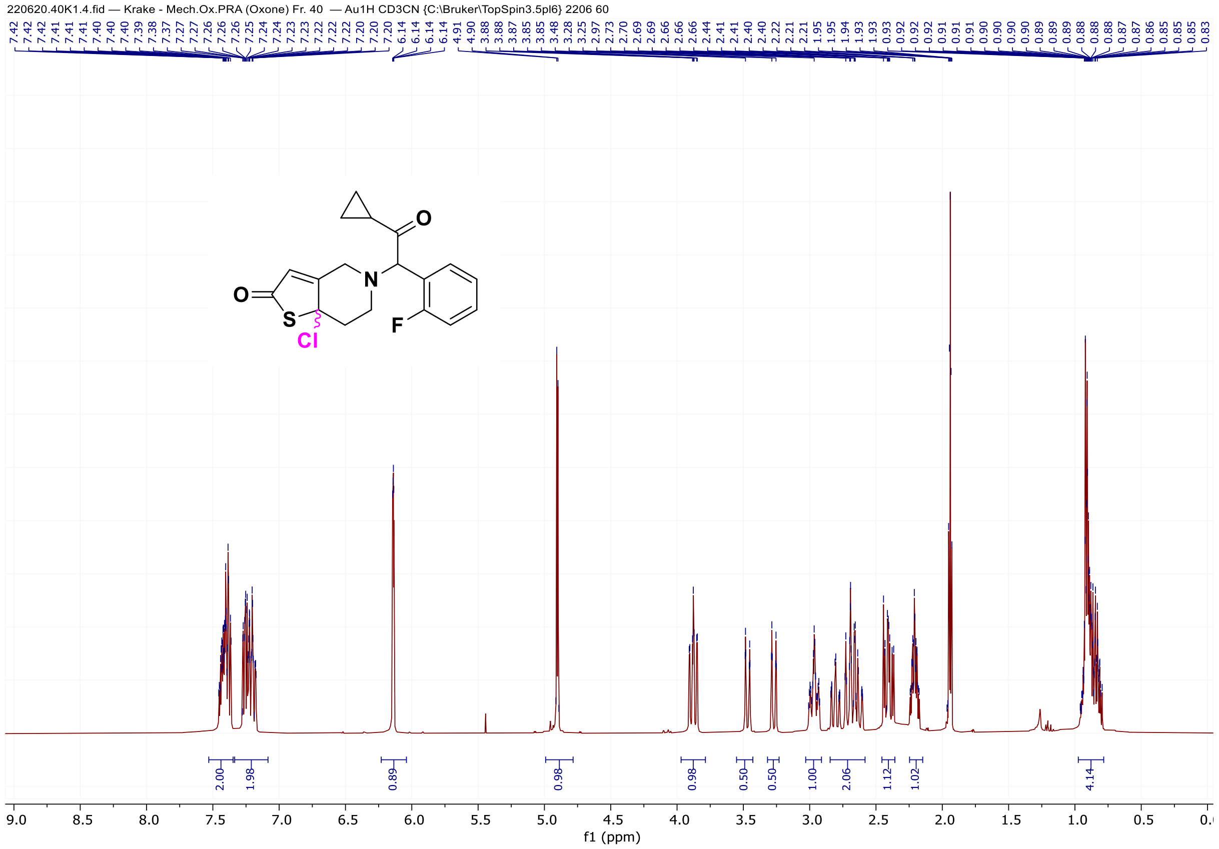

Figure S24. ^{1}H NMR spectrum of halogenated product **PRA-DP-4** (CD_3CN, 400 MHz).

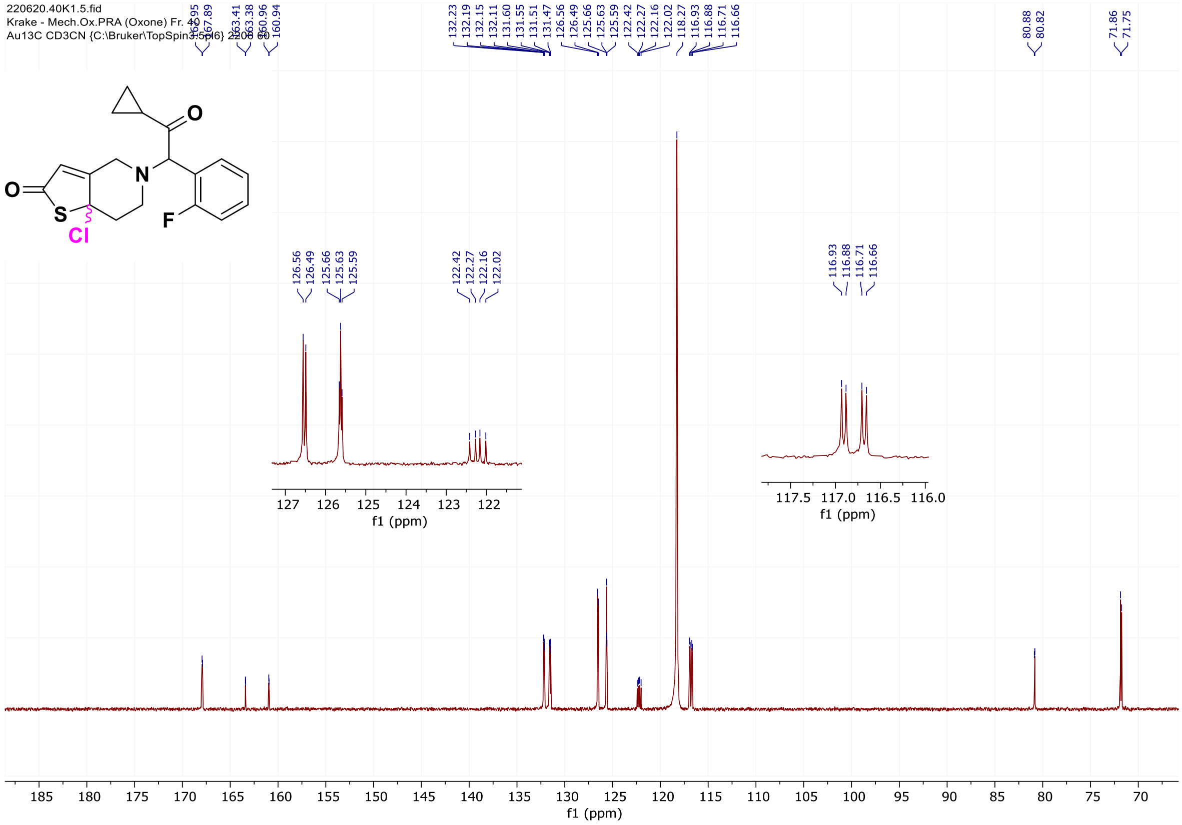

Figure S25. ^{13}C NMR spectrum of halogenated product **PRA-DP-4** (CD_3CN, 101 MHz).

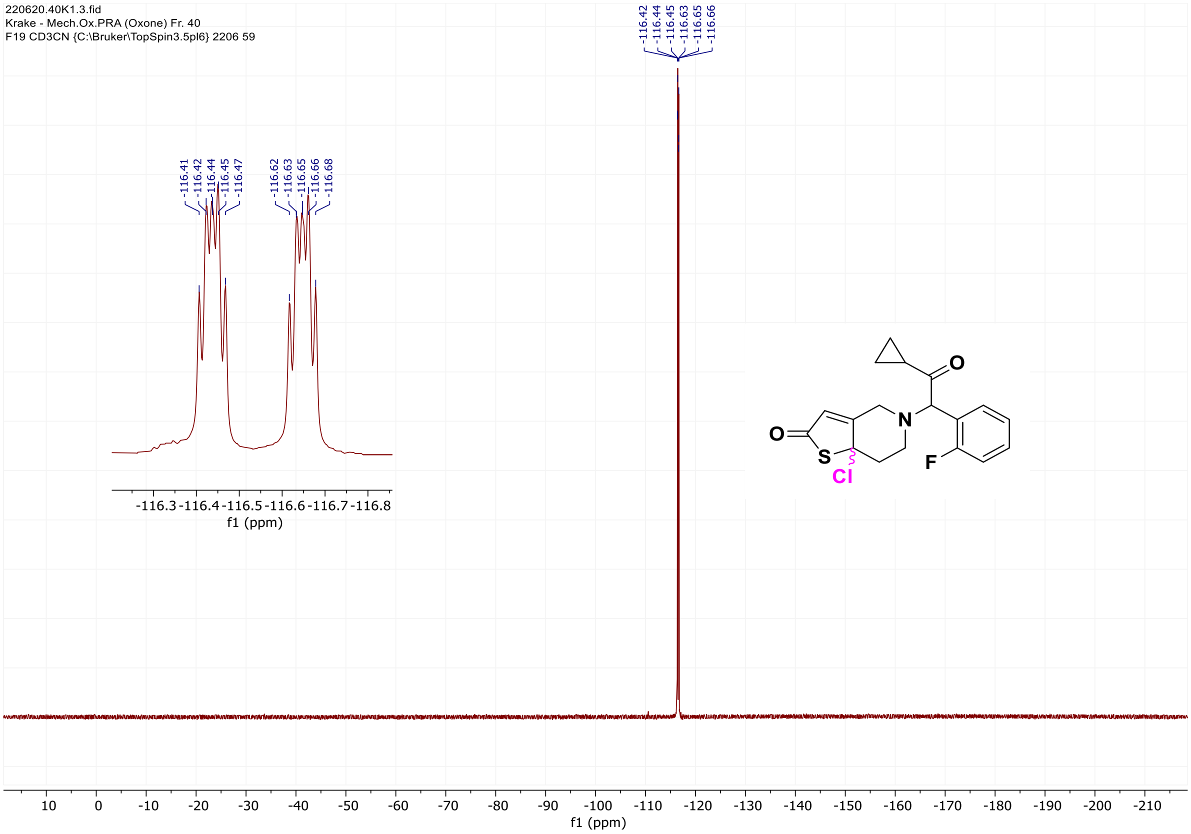

Figure S26. ^{19}F NMR spectrum of halogenated product **PRA-DP-4** (CD_3CN, 376 MHz).

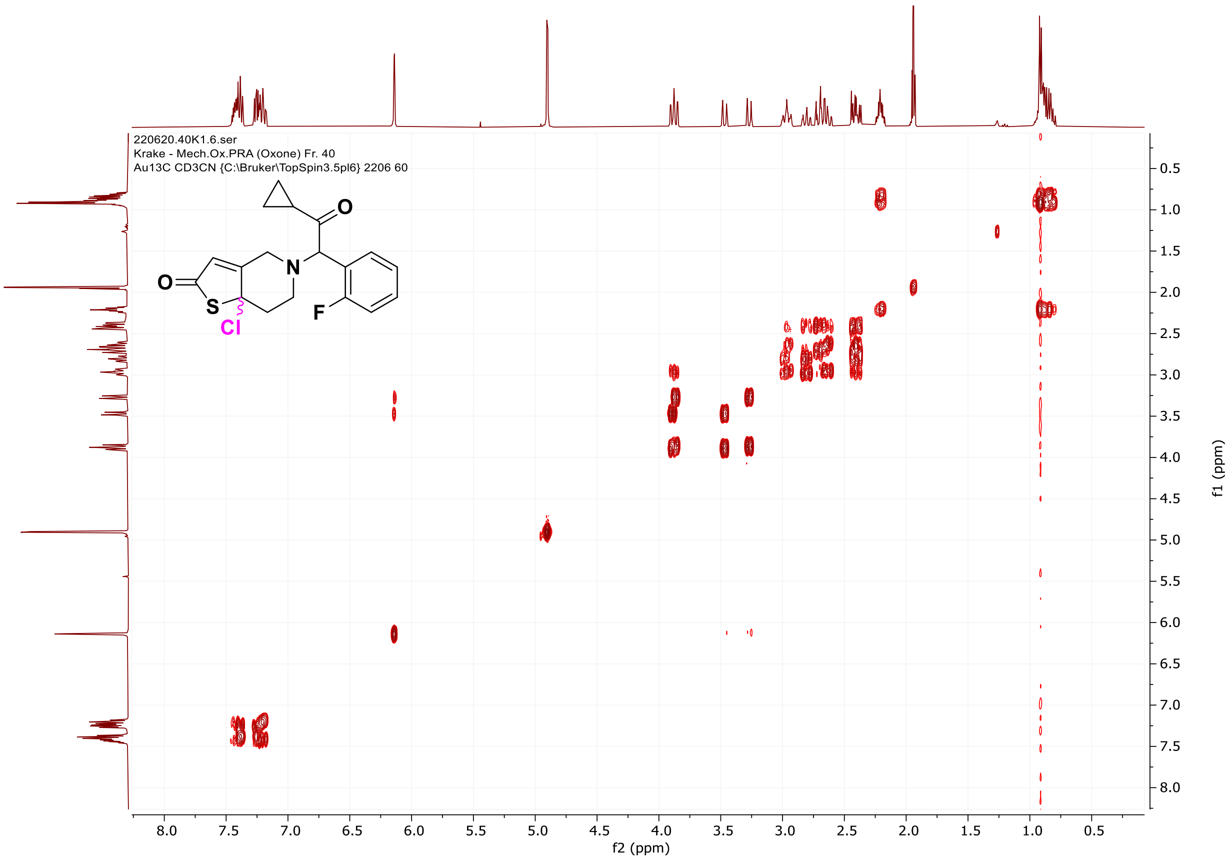

Figure S27. $^{1}H/^{1}H$ COSY spectrum of halogenated product **PRA-DP-4** (CD_3CN, 400 MHz).

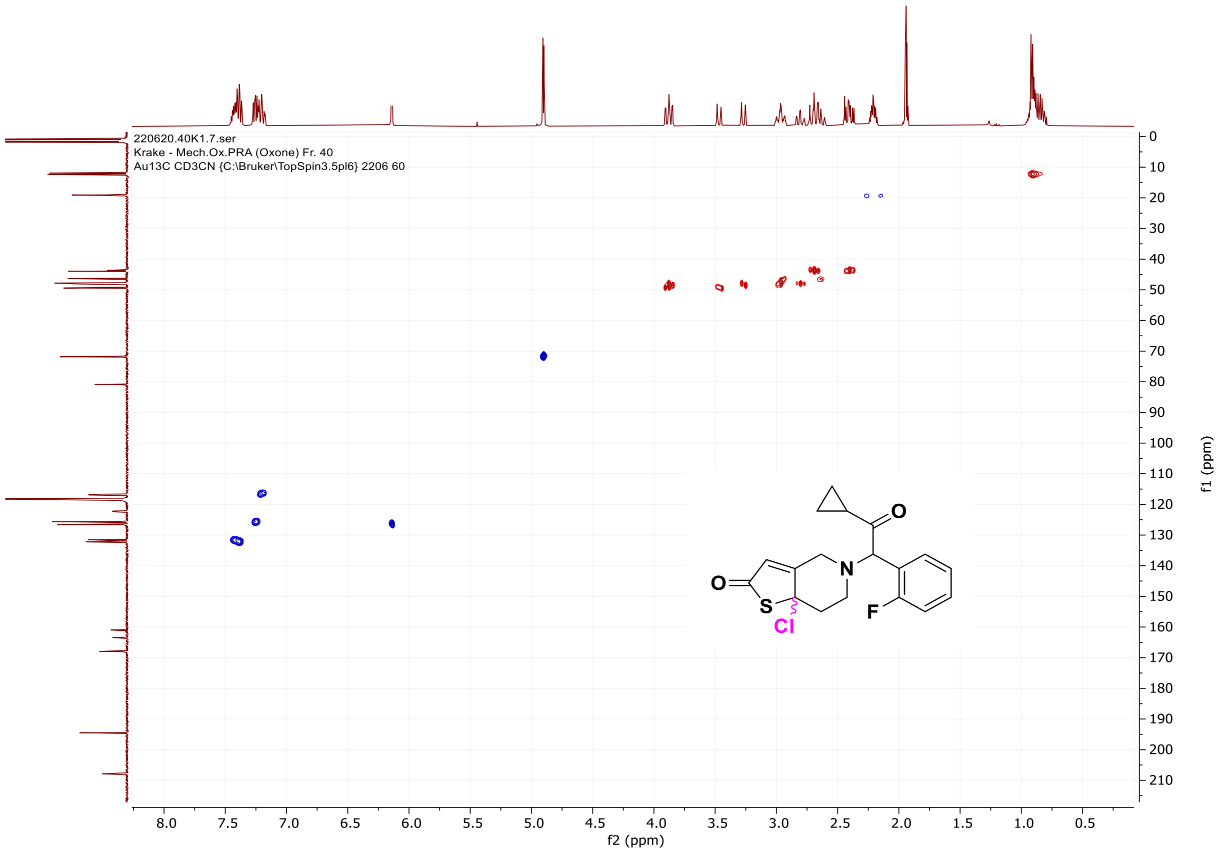

Figure S28. $^{1}H/^{13}C$ HSQC-edited spectrum of halogenated product **PRA-DP-4** (CD_3CN, 400 MHz).

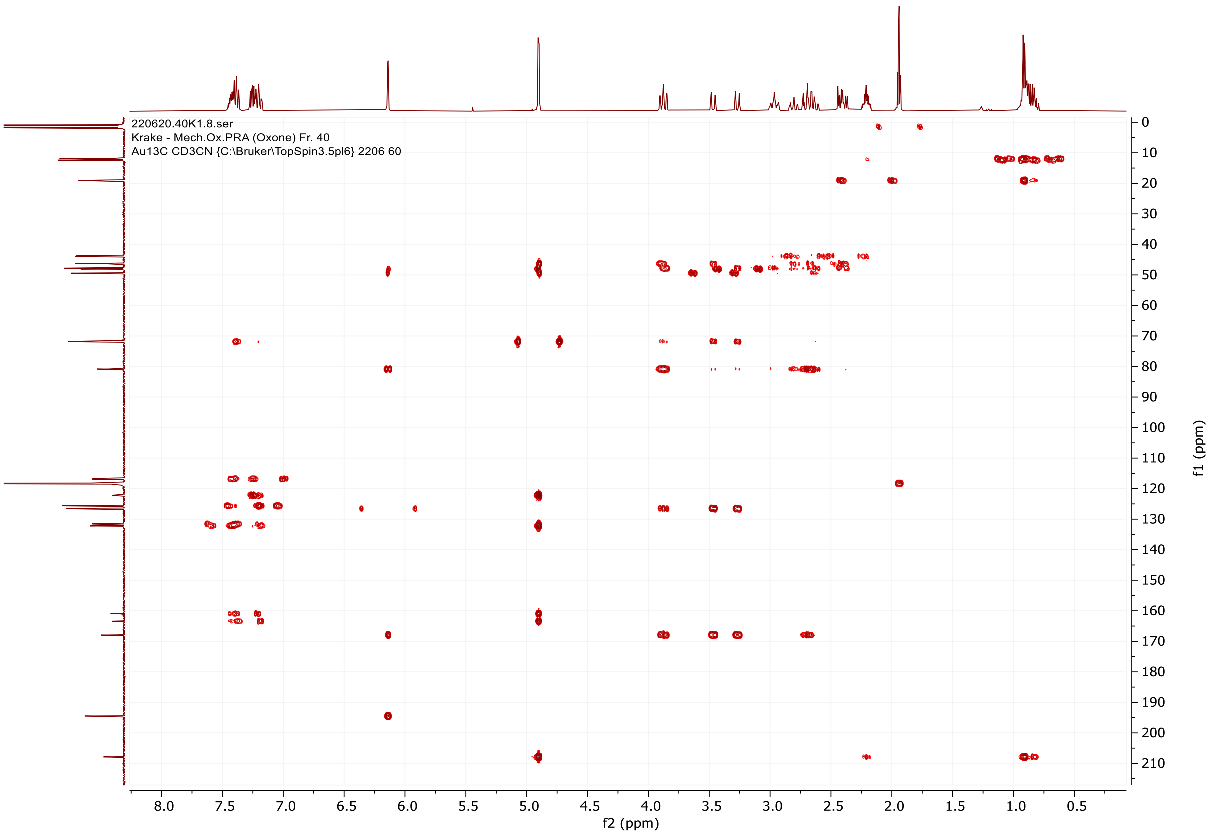

Figure S29. $^{1}H/^{13}C$ HMBC spectrum of halogenated product **PRA-DP-4** (CD_3CN, 400 MHz).

Table S7. ^{1}H and ^{13}C assignments for **PRA-DP-4** (X = Cl) product.

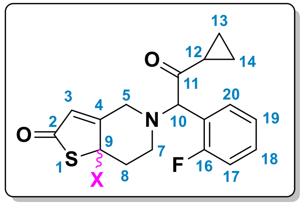

Position	X = H, Baertschi OXTP-Dia 1[b]		X = H, Baertschi OXTP-Dia 2[b]		X = Cl, Diastereomer 1[a]		X = Cl, Diastereomer 2[a]	
	^{1}H	^{13}C	^{1}H	^{13}C	^{1}H or ^{19}F	^{13}C ($J_{C,F}$)	^{1}H or ^{19}F	^{13}C ($J_{C,F}$)
1 (S)								
2 (C=O)		199.3		199.8		194.44		194.44
3 (CH)	6.10	126.0	6.14	126.2	6.14	126.47	6.14	126.53
4 (C)		170.44		171.0		167.86		167.93
5 (CH_2)	2.90/3.94	51.5	3.11/4.01	53.2	3.27/3.86	48.05	3.47/3.89	49.39
6 (N)								
7 (CH_2)	2.56/3.13	50.7	2.34/3.08	49.0	2.63/2.95	46.31	2.81/2.98	47.76
8 (CH_2)	1.80/2.49	34.8	1.78/2.44	34.6	2.39/2.71	43.67	2.42/2.67	43.93
9 (C–X)	4.32	51.5	4.32	51.6		80.79		80.85
10 (CH)	4.89	71.8	4.88	71.9	4.90	71.72	4.91	71.84
11 (C=O)		207.9		208.5		207.79		207.84
12 (CH)	2.30	18.4	2.28	?	2.21	19.03	2.22	19.11
13 (CH_2)	0.94	11.8	0.95	11.9	0.83/0.93	11.88	0.83/0.93	11.96
14 (CH_2)	0.94	11.8	0.95	11.9	0.88	12.29	0.88	12.37
15 (C_{Ar})		121.9		122.6		122.1 (?)		122.3 (?)
16 (C–F)		161.7		162.5	–116.5	162.14 (246)	–116.7	162.16 (246)
17 ($C_{Ar}H$)	7.26	116.3	7.26	116.4	7.20	116.74 (23)	7.20	116.79 (23)
18 ($C_{Ar}H$)	7.47	131.1	7.47	131.2	7.42	131.49 (8)	7.42	131.53 (9)
19 ($C_{Ar}H$)	7.31	125.1	7.30	125.3	7.25	125.58 (3)	7.25	125.62 (3)
20 ($C_{Ar}H$)	7.43	131.8	7.43	131.9	7.38	132.13 (8)	7.38	132.16 (8)

Note: Positions show the number on the chemical structure above. Chemical shifts (δ) are reported in ppm relative to TMS. *J*-values are shown in Hz in parentheses.

(a) ^{1}H (400 MHz, CD_3CN), ^{13}C (101 MHz) and ^{19}F NMR (376 MHz) signals correspond to an equimolar mixture of diastereomers, an assignment to the individual compounds has not been done (*i.e.,* occurrence of numbers in the same column does not necessarily mean that the signals belong to the same diastereomer)! Due to small diastereomeric dispersion, ^{1}H signals for several positions could not be separated, and only one average value for both isomers is given. Assignments to positions 13 and 14 may be interchanged. C–F coupling constants for position 15 are not given, because it was not possible to determine those without ambiguity.

(b) ^{1}H (CD_3CN at 0 °C) and ^{13}C NMR shift values from Baertschi *et al.*[4] for comparison purpose: another pair of diastereomeric degradation products with the same structure and strikingly similar spectroscopic data.

HRMS data

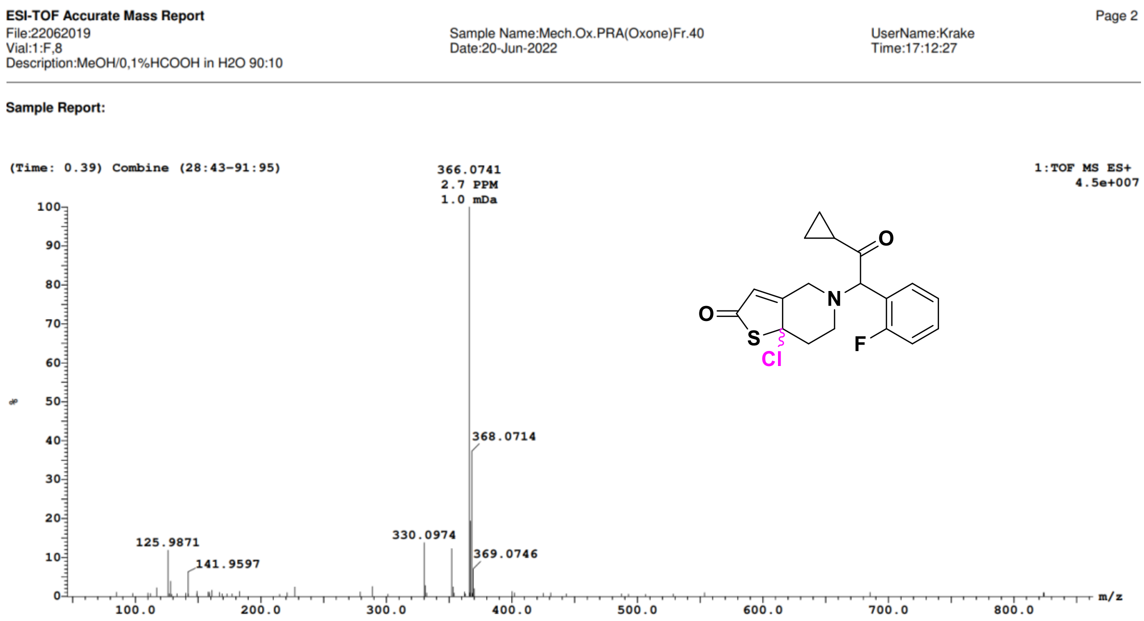

Figure S30. HRMS data of halogenated product **PRA-DP-4** ($C_{18}H_{18}ClNO_2FS^+$, calc. $[M]^+$: 366.0731 found: 366.0741; 2.7 ppm error).

References

1 ^{1}H-NMR (300 MHz, $CDCl_3$) δ 1.79-1.93 (m, 1H), 2.30-2.40 (m, 1H), 2.56-2.70 (m, 1H), 3.00-3.27 (m, 2H), 3.72 (s, 3H), 3.79-3.93 (m, 1H), 4.12-4.19 (m, 1H), 4.89 (d, 1H, J=5.6 Hz), 6.00 (d, 1H, J=5.2 Hz), 7.26-7.50 (m, 4H); ^{13}C-NMR (75 MHz, $CDCl_3$) δ 33.9, 34.0, 49.0, 49.7, 51.1, 51.6, 52.2, 52.4, 67.3, 76.6, 77.0, 77.4, 126.6, 126.8, 127.2, 129.8, 130.1, 132.7, 134.8, 167.2, 167.4, 170.8, 198.6.

2 Shan, J.; Zhang, B.; Zhu, Y.; Jiao, B.; Zheng, W.; Qi, X.; Gong, Y.; Yuan, F.; Lv, F.; Sun, H. Overcoming Clopidogrel Resistance: Discovery of Vicagrel as a Highly Potent and Orally Bioavailable Antiplatelet Agent. *J. Med. Chem.* **2012**, *55*, 3342-3352. DOI: 10.1021/jm300038c.

3 Liu, C.; Chen, Z.; Zhong, K.; Li, L.; Zhu, W.; Chen, X.; Zhong, D. Human Liver Cytochrome P450 Enzymes and Microsomal Thiol Methyltransferase Are Involved in the Stereoselective Formation and Methylation of the Pharmacologically Active Metabolite of Clopidogrel. *Drug Metab. Dispos.* **2015**, *43*, 1632. DOI: 10.1124/dmd.115.064949.

4 Baertschi, S. W.; Maxwell-Backer, L.; Clemens, M.; Smitka, T. A.; Draper, J. R.; Taylor, K. W.; Kaerner, A.; Jansen, P. J. The Degradation Chemistry of Prasugrel Hydrochloride: Part 1—Drug Substance. *J. Pharm. Sci.* **2019**, *108*, 2842-2857. DOI: https://doi.org/10.1016/j.xphs.2019.04.008.
